# O-GlcNAcylation: A molecular switch linking brain health to neurodegeneration

**DOI:** 10.4103/NRR.NRR-D-25-00101

**Published:** 2025-09-03

**Authors:** Nan Shao, Xiaoyan Zhang, Yunzhi Ge, Jiaxuan Tang, Huawu Gao, Wenwen Si, Biao Cai

**Affiliations:** 1 College of Integrated Chinese and Western Medicine, Anhui University of Chinese Medicine, Hefei, Anhui Province, China; 2 Department of Rehabilitation, the First Affiliated Hospital, Anhui University of Chinese Medicine, Hefei, Anhui Province, China; 3 Institute of Integrated Chinese and Western Medicine, Anhui Academy of Chinese Medicine, Hefei, Anhui Province, China

**Keywords:** Alzheimer’s disease, amyotrophic lateral sclerosis, Huntington’s disease, neural regeneration, neurodegeneration, neuronal function, O-GlcNAcase, O-linked N-acetylglucosamine, O-linked N-acetylglucosamine transferase, Parkinson’s disease

## Abstract

Neurodegenerative disorders are typically caused by harmful protein accumulation and nerve cell damage. A post-translational modification called O-linked N-acetylglucosamine ylation acts as a critical regulator in these disorders by controlling protein behavior, cell signaling, and energy balance. This modification is dynamically balanced through the cooperative actions of O-linked N-acetylglucosamine transferase and O-GlcNAcase. In healthy brains, O-GlcNAcylation supports nerve cell function and survival, but its imbalance contributes to disease progression. Notably, the effects of O-GlcNAcylation differ across disorders. This review reveals how O-GlcNAcylation bridges molecular mechanisms to neurodegeneration, as well as the prospects of targeted O-linked N-acetylglucosamine acylation therapy for neurodegenerative diseases. In Alzheimer’s disease, it blocks toxic changes in key proteins like tau and amyloid-beta. In Parkinson’s disease, it reduces the clumping of alpha-synuclein, yet may disrupt dopamine production. In amyotrophic lateral sclerosis, it protects nerve fiber transport systems. Additionally, O-GlcNAcylation plays an indispensable part in other neurodegenerative conditions, including Huntington’s disease, aging, Machado-Joseph disease, multiple sclerosis, and giant axonal neuropathy. New therapies targeting this mechanism include glucosamine supplements and O-GlcNAcase inhibitors, which show clinical promise but face translational challenges.

## Introduction

Neurodegenerative diseases are a group of complex disorders characterized by progressive neuronal degeneration and functional impairment, primarily including Alzheimer’s disease, Parkinson’s disease, amyotrophic lateral sclerosis, and Huntington’s disease (Phillips and Picard, 2024). Globally, Alzheimer’s disease accounts for approximately 60%–70% of dementia cases, while Parkinson’s disease affects over 10 million people, with morbidity increasing significantly alongside the aging population (Nasb et al., 2024; Tinazzi et al., 2025). As a major public health challenge in aging societies, elucidating the pathogenesis of neurodegenerative diseases becomes paramount for advancing valid disease-modifying therapies. Studies have indicated that the core pathological hallmark of these diseases is abnormal protein aggregation, such as amyloid-beta (Aβ) plaques and phosphorylated tau protein in Alzheimer’s disease (Teunissen et al., 2025), alpha-synuclein (α-syn) protein in Parkinson’s disease (Endo et al., 2024), and transactive response DNA-binding protein 43 (TDP-43) protein in amyotrophic lateral sclerosis (Irwin et al., 2024). These pathologies are also accompanied by neuronal loss, synaptic dysfunction, and neuroinflammation (Dejanovic et al., 2024). Thus, targeting the elimination of abnormal protein accumulation becomes a promising therapeutic strategy for neurodegenerative diseases.

O-linked N-acetylglucosamine (O-GlcNAc) modification represents a dynamic regulatory process prevalent in eukaryotic cells, characterized by the conjugation of N-acetylglucosamine (GlcNAc) to hydroxyl groups on target proteins through an O-glycosidic bond (Huo et al., 2022). O-GlcNAcylation has been widely explored for its involvement in diverse cellular processes, including protein stabilization (Kweon et al., 2024), signal transduction (Narayanan et al., 2023), transcriptional regulation (Xie et al., 2021), and cell cycle control (Zhao et al., 2024). The O-GlcNAcylation is tightly linked to cellular glucose metabolism, serving as a dynamic regulator of energy homeostasis (Wang et al., 2024). Analogous to phosphorylation, O-GlcNAcylation exists on serine (Ser) and threonine (Thr) of nuclear and cytoplasmic proteins, conferring additional functional complexity to proteins (Vosseller et al., 2001). Notably, O-GlcNAcylation not only plays essential roles in neuronal physiology but is also implicated in pathological conditions, particularly neurodegenerative diseases (Pratt and Vocadlo, 2023).

O-GlcNAcylation is highly concentrated in the nervous system and has been identified on hundreds of proteins, including amyloid precursor protein (APP), tau, α-syn, and TDP-43, which are involved in the neurodegenerative progression (Ryan et al., 2019). Consequently, it is essential to elucidate the biological significance of O-GlcNAcylation in neurodegeneration. This review summarized the regulatory mechanisms of O-GlcNAcylation, its effect on neuronal homeostasis, and emerging evidence linking this modification to various neurodegenerative disorders. The synthesis aimed to provide novel insights for developing targeted therapeutic interventions against these progressive diseases.

## Search Strategy

The literature cited in this review was obtained through searches conducted on the PubMed database (biomedical literature). The search period spanned from January 1984 to May 2025. The search in all fields included the following search terms and their combinations: “O-GlcNAc,” “O-linked N-acetylglucosamine,” “O-GlcNAc transferase,” “O-GlcNAcase,” “O-GlcNAcylation,” “hexosamine biosynthetic pathway,” “neurons,” “neurodegenerative diseases,” “Alzheimer disease,” “Parkinson disease,” “amyotrophic lateral sclerosis,” “Huntington disease,” “tauopathies,” “alpha-synuclein,” “TDP-43,” “neurofibrillary tangles,” “amyloid beta,” and “dopamine.” Literature was further screened based on titles and abstracts. Studies that were not neurologically relevant and did not involve O-GlcNAc-related mechanisms were excluded. Ultimately, original research of direct relevance to this review purpose was included to examine the role of O-GlcNAcylation in neurodegenerative diseases.

## Brief History of O-GlcNAcylation

The discovery of O-GlcNAcylation dates back to 1984 when Gerald W. Hart’s team first identified this modification in the proteins of lymphocytes (Torres and Hart, 1984). In 1986, Holt further revealed the widespread distribution of O-GlcNAc across cellular compartments, with notable enrichment in the nucleus and cytoplasm (Holt and Hart, 1986). By 1987, Ser and Thr residues were established as the primary sites of O-GlcNAcylation (Holt et al., 1987). The purification of O-GlcNAc transferase (OGT) in 1992 (Haltiwanger et al., 1992) and the subsequent cloning of its gene in 1997 (Kreppel et al., 1997) unveiled its unique glycosyltransferase properties. Dong and Hart (1994) isolated O-GlcNAcase (OGA) from the rat spleen, distinguishing it from lysosomal hexosaminidases. In 2001, Hart’s team successfully cloned OGA, promoting research on the dynamic reversibility of O-GlcNAcylation (Gao et al., 2001). In 2002, Alloxan emerged as the first reported OGT inhibitor (Konrad et al., 2002), followed by the development of NAG-thiazoline, a highly selective OGA inhibitor, by Vocadlo’s group in 2005 (Macauley et al., 2005), which propelled research into O-GlcNAc signaling regulation. Recent innovations in mass spectrometry and proteomics permitted the systematic location of O-GlcNAcylation sites, uncovering their multifaceted roles in epigenetic regulation and metabolic stress responses (Xu et al., 2021b). Since its discovery, O-GlcNAcylation has been demonstrated to regulate diverse cellular processes. However, its dysregulation is closely associated with pathology such as cancer, immune disorders, diabetes, and neurodegenerative diseases (Cheng et al., 2024a).

**Figure 1:**
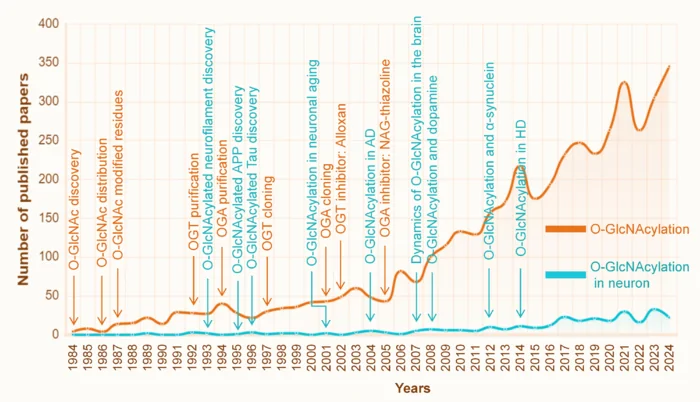
Historical evolution in O-GlcNAcylation research. Bibliometric analysis of PubMed-indexed publications (1984–2024) reveals a progressive increase in research output related to O-GlcNAcylation and neuronal O-GlcNAcylation. Created with Word Processing System Presentation (version 12.1.0.19302). AD: Alzheimer’s disease; HD: Huntington’s disease; O-GlcNAc: N-acetylglucosamine; OGA: O-GlcNAcase; OGT: O-GlcNAc transferase.

Research on the role of O-GlcNAcylation in neuronal function and neurodegenerative diseases has evolved constantly. In 1993, O-GlcNAcylation was found to specifically localize to neurofilament proteins, modulating their assembly and laying the groundwork for its implication in amyotrophic lateral sclerosis pathogenesis (Dong et al., 1993). Subsequent studies in 1995 and 1996 identified extensive O-GlcNAcylation on APP and tau protein respectively, suggesting a potential link to Alzheimer’s disease (Griffith et al., 1995; Arnold et al., 1996). By 2001, O-GlcNAcylation was shown to participate in neuronal development, maturation, and aging (Rex-Mathes et al., 2001). A pivotal study in 2004 revealed significantly reduced O-GlcNAcylation levels of tau protein in Alzheimer’s disease brains, inversely correlating with hyperphosphorylation and implicating this modification in suppressing neurofibrillary tangle formation (Robertson et al., 2004). In 2007, quantitative proteomics first demonstrated that O-GlcNAc undergoes reversible dynamic regulation in response to synaptic activity, mediating neuronal communication (Khidekel et al., 2007). Direct evidence linking O-GlcNAcylation to Parkinson’s disease emerged in 2008 and 2012, with findings that dopamine secretion and α-syn aggregation are modulated by this modification (Bork et al., 2008; Marotta et al., 2012). In 2014, O-GlcNAcylation was further implicated in regulating neuronal survival in Huntington’s disease models, expanding its theoretical relevance to neurodegenerative disorders (Kumar et al., 2014). The developmental milestones of O-GlcNAcylation in neurodegenerative diseases are summarized in **Figure 1**.

## Process of O-GlcNAcylation

Disturbed glucose metabolism is recognized as a feature of neurodegeneration and cerebral impairment. Within glucose metabolic pathways, dysregulation of the hexosamine biosynthetic pathway (HBP) and its associated O-GlcNAc cycle has been demonstrated to critically contribute to the pathological mechanisms underlying neurodegenerative diseases (Kim et al., 2023). Research has shown that during mammalian development and adulthood, the overall protein O-GlcNAcylation in the brain gradually decreases (Liu et al., 2012). Furthermore, the O-GlcNAc cycle is dynamically coordinated by two key enzymes: OGT, which catalyzes the addition of GlcNAc moieties, and OGA, responsible for their removal.

### Origin of O-GlcNAcylation: hexosamine biosynthetic pathway

HBP is an amino sugar metabolic pathway primarily involved in nucleoside sugar synthesis, such as uridine diphosphate-N-acetylglucosamine (UDP-GlcNAc), which is crucial for the synthesis and modification of various biomolecules (Grilo et al., 2024; Liu et al., 2024). The actual steps are as follows. (I) Glucose phosphorylation: Upon entering the neuron, glucose is phosphorylated by hexokinase and adenosine 5′-triphosphate (ATP), converting it into glucose-6-phosphate. (II) Isomerization: Glucose-6-phosphate is isomerized to fructose-6-phosphate by glucose-6-phosphate isomerase (Abbas et al., 2016; Karlstaedt et al., 2020). (III) Amination: Fructose-6-phosphate binds to glutamine and undergoes a reaction catalyzed by glutamine fructose-6-phosphate amidotransferase to make glucosamine-6-phosphate (Oikari et al., 2016). (IV) Acetylation: Glucosamine-6-phosphate acetyltransferase catalyzes the addition of acetyl-CoA to glucosamine-6-phosphate, forming N-acetylglucosamine-6-phosphate (Alberione et al., 2024). (V) Isomerization: N-acetylglucosamine-6-phosphate catalyzes the generation of N-acetylglucosamine-1-phosphate via N-acetylglucosamine phosphoglucomutase (Nishitani et al., 2006; Raimi et al., 2018). (VI) Pyrophosphorylation: N-acetylglucosamine-1-phosphate and uridine triphosphate produce UDP-GlcNAc in the presence of N-acetylglucosamine pyrophosphorylase (Raimi et al., 2020).

**Figure 2:**
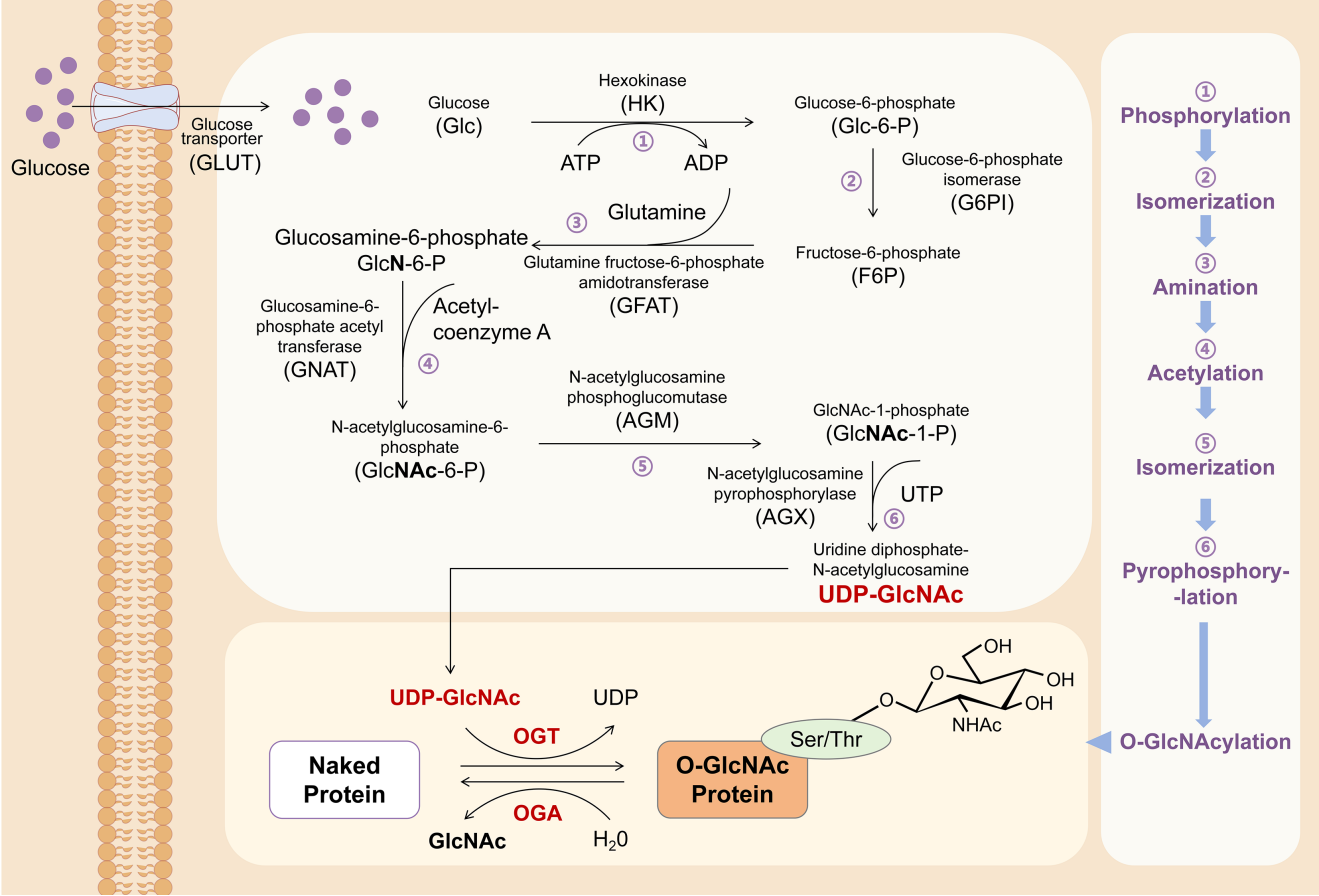
HBP and O-GlcNAc cycle in neurons. After glucose enters the neuron, it undergoes a series of processes, including phosphorylation, isomerization, amination, acetylation, allosteric effects, and pyrophosphorylation, to generate UDP-GlcNAc. OGT covalently attaches GlcNAc from UDP-GlcNAc to the protein, and OGA hydrolyzes GlcNAc off the protein to form the O-GlcNAc cycle. Created with Word Processing System Presentation (version 12.1.0.19302). ADP: Adenosine diphosphate; ATP: adenosine triphosphate; HBP: hexosamine biosynthetic pathway; OGA: O-GlcNAcase; O-GlcNAc: N-acetylglucosamine; OGT: O-GlcNAc transferase; UDP: uridine diphosphate; UTP: uridine triphosphate.

UDP-GlcNAc serves as the exclusive donor substrate for O-GlcNAcylation. Through OGT-mediated catalysis, GlcNAc is covalently attached to Ser or Thr residues of target proteins, forming O-GlcNAcylation (Li et al., 2021; Song et al., 2022). This reversible process, counterbalanced by OGA-mediated hydrolysis, is a key modulator in orchestrating neuronal signaling and homeostatic regulation (Fan et al., 2021). The specific process of the UDP-GlcNAc and O-GlcNAc cycle is shown in **Figure 2**.

### Writer of O-GlcNAcylation: O-GlcNAc transferase

**Figure 3:**
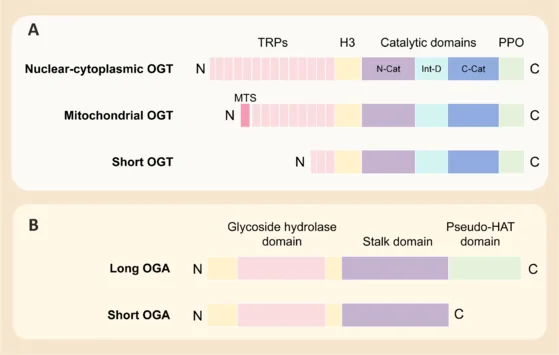
Schematic protein structures of O-GlcNAcylation-modified enzymes. (A) Protein structure diagram of the three OGT isoforms. (B) Protein structure diagram of the two OGA isoforms. Created with Word Processing System Presentation (version 12.1.0.19302). C-Cat: C-terminal catalytic domain; HAT: histone acetyltransferase; Int-D: intervening domain; MTS: mitochondrial targeting sequence; N-Cat: N-terminal catalytic domain; OGA: O-GlcNAcase; OGT: O-GlcNAc transferase; O-GlcNAc: N-acetylglucosamine; PPO: phosphoinositide-binding domain; TRPs: tetratricopeptide repeats.

OGT, the key enzyme catalyzing protein O-GlcNAcylation, is controlled by its isoform diversity, structural features, and dynamic regulatory mechanisms. Although ubiquitously expressed, OGT is particularly enriched in brain tissue (Okuyama and Marshall, 2003). Structurally, OGT contains an N-terminal tetratricopeptide repeat (TPR) domain essential for substrate recognition and a C-terminal glycosyltransferase family B domain responsible for catalytic activity (Lazarus et al., 2011), connected by an intervening helix H3. The alternative splicing generates three primary isoforms in humans as follows (Zhang et al., 2022). (I) Nuclear-cytoplasmic OGT, comprising 13.5 TPRs, is located in the nucleus and cytoplasm, mediating transcriptional repression and stress responses (Love et al., 2003; Trapannone et al., 2016). (II) Mitochondrial OGT, featuring 9.5 TPRs and an N-terminal mitochondrial targeting sequence, is resided in the mitochondrial inner membrane, regulating redox homeostasis (Sacoman et al., 2017). (III) Short OGT, containing only 2.5 TPRs, is distributed in the cytoplasm and nucleus, modulating cell cycle progression and proliferation (Liu et al., 2019). All isoforms share a conserved C-terminal catalytic domain and an intervening domain, which governs substrate specificity and protein-protein interactions (Blankenship et al., 2023). The C-terminal domain also harbors a phosphoinositide-binding domain, facilitating membrane recruitment in response to insulin signaling (Yang et al., 2008). Structural studies indicated that the dimeric structure of OGT (Meek et al., 2021) and missense mutations in its catalytic domain collectively provide the structural basis of intellectual-related diseases (Pravata et al., 2019; Pravata et al., 2020). The protein structure of OGT is shown schematically in **Figure 3A**.

### Eraser of O-GlcNAcylation: O-GlcNAcase

OGA, the sole known glycosidase responsible for removing O-GlcNAcylation (Wu et al., 2024), is enriched in skeletal muscle, pancreas, and brain (Lambert et al., 2018). OGA forms two main subtypes through alternative splicing, including long OGA and short OGA (Kim et al., 2007). Long OGA comprises three functional domains (King et al., 2019). (I) An N-terminal glycoside hydrolase catalytic domain adopting a classical (β/α)_8_ triose-phosphate isomerase barrel folding, responsible for hydrolyzing O-GlcNAcylation. (II) A central helical stalk domain, consisting of four α-helices, stabilizes the catalytic core and contributes to substrate recognition. (III) A C-terminal pseudo-histone acetyltransferase domain, potentially mediating protein interactions. In contrast, short OGA lacks the histone acetyltransferase domain, resulting in markedly reduced enzymatic activity (Keembiyehetty et al., 2011). The protein structure of OGA is shown schematically in **Figure 3B**. Furthermore, crystal structure analyses have suggested that human OGA assembles into a flexible dimer characterized by α-helix swapping, with its active pocket located within a V-shaped cleft formed by the C-terminal helical bundle and catalytic domains (Elsen et al., 2017; Roth et al., 2017).

Consequently, the dynamics of O-GlcNAcylation on substrates have critical importance in the maintenance of neuronal status and the regulation of protein functions. OGT and OGA jointly regulated the O-GlcNAc cycle and interacted with each other. In the complex OGT-OGA structure, a flexible OGA fragment occupied the extended substrate-binding groove of OGT and positioned a Ser residue for O-GlcNAcylation, thus hindering OGT from binding to other substrates (Lu et al., 2023). The GlcNAc electrophilic probe has revealed that the catalytic domain and stem domain of OGT are key to its interaction with OGA (Kositzke et al., 2021). Functionally, disturbances in the OGT/OGA induced deficits in learning, memory, and other neurobehavioral functions (Sung et al., 2024). The neuronal developmental and functional deficits observed in OGT-mutant Drosophila can be rescued in part by genetic or chemical targeting of OGA (Czajewski et al., 2024). This evidence suggested that the normal nervous system required an equilibrium of OGT and OGA.

## O-GlcNAcylation in Normal Brain

Both OGA and OGT transcripts are highly expressed in the brain, with peak abundance observed in Purkinje cells and hippocampal neurons (Liu et al., 2004). The highly enriched O-GlcNAcylation plays multifaceted and critical roles in neuronal functional regulation, encompassing neurogenesis, development, apoptosis, signal transduction, and energy metabolism (Hwang and Rhim, 2018). Therefore, modulating O-GlcNAcylation is essential for maintaining neuronal homeostasis and functionality.

### Generation and development of neurons

Generally speaking, the neuron generation usually commences in the early stage of embryogenesis. Initially, this process is carried out through the self-renewal and differentiation ability of neural stem cells (NSCs) (Tanaka et al., 2020). An upregulation of total O-GlcNAcylation levels has been correlated with reduced proliferation of NSCs, premature differentiation of cortical neurons, and defects of various regulatory factors during cortical development (Parween et al., 2017). During the neuronal differentiation of human embryonic stem cells (ESCs), O-GlcNAcylation levels activated the gene level of key neurogenic transcription factors, among which were *EOMES*, *TBR1*, *HOPX*, *NEUROD2/4/6*, and *NEUROG1/2* (Parween et al., 2022). Moreover, disturbances in OGT and the protein O-GlcNAcylation have been shown to expedite the differentiation of human ESCs along the neuronal lineage (Andres et al., 2017). A recent chemical proteomics approach identified 979 O-GlcNAcylated proteins and 1340 modified sites in mouse ESCs. Notably, the O-GlcNAc stoichiometry in 123 sites of 83 proteins exhibited a decreasing trend during neuronal lineage differentiation (Hao et al., 2023).

After being generated, neurons are required to migrate to appropriate locations and then differentiate into mature states with specific capacities, including the development of cell bodies, axons, and dendrites, and ultimately the formation of functional neural networks (Suciu and Caspary, 2021). Downregulation of O-GlcNAcylation, resulting from the OGT inhibition, led to a series of severe defects. These included the drastic contraction of cortical and hippocampal tissue structures, as well as the suppression of dendritic and axonal differentiation in neurons (Shen et al., 2021). Moreover, the decreased O-GlcNAcylation impaired the proliferation of neural ESCs and hindered the migration of newborn neurons, thus affecting the dendritic development of hippocampal neurons and the maturation of spines (Cheng et al., 2022). Research indicated that this impairment in hippocampal neuron development arose from O-GlcNAcylation disruption at the Thr203 site of the human X-linked methyl-CpG-binding protein 2 (Cheng et al., 2020).

O-GlcNAcylation is capable of regulating specific nerve cell structures and functions during neurodevelopment, thereby maintaining complex activities such as sensory perception, motor coordination, and cognition. For example, Purkinje cells are a special kind of neuron exported from the cerebellar cortex, in which the absence of OGT induces cerebellar developmental defects such as severe ataxia in mice (Liu et al., 2023). OGT also regulated neurogenesis in granule cell precursors, which were vital for cerebellar growth. This role was mediated by the OGT-catalyzed O-GlcNAcylation at the Ser355 site of GLI family zinc finger 2, which subsequently activated the Sonic hedgehog signaling pathway (Chen et al., 2022). Besides, O-GlcNAcylation was implicated in regulating the length of the neuronal primary cilium. Elevated O-GlcNAc induced the assembly of longer primary cilia but ultimately led to premature neuronal development (Tian et al., 2023). As neurons mature, the function of the nervous system is gradually refined, a process that continues into adulthood, especially in the development of cognitive functions (Babcock et al., 2021). OGT directly bound to Notch1 and promoted its O-GlcNAcylation, which was degraded by OGT deficiency, exhibiting reduced adult NSC pools, neurogenesis abnormality, and cognitive damage in adult mice (Chen et al., 2021). However, the blockage of O-GlcNAcylation of the transcription factor cyclic adenosine monophosphate-response element binding protein altered cellular physiology and behavioral capacity, promoted axonal and dendritic development, and consolidated long-term memory (Rexach et al., 2012).

In summary, O-GlcNAcylation plays a pivotal role in both embryonic and postnatal neurodevelopmental processes (Mitchell et al., 2022). Hippocampal neurogenesis and neural development persist throughout human physiological and pathological aging but decline with age and are significantly impaired in neurodegenerative diseases (Moreno-Jiménez et al., 2019; Abramov, 2022). Therefore, maintaining normal neurogenesis and development through O-GlcNAcylation homeostasis is critical for preventing and alleviating neurodegenerative damage.

### Damage and death of neurons

Overexpression of OGT in the mouse brain to enhance O-GlcNAcylation can mitigate diverse neuropathological outcomes, including cellular morphological damage, neuronal death, and motor deficits (Kim et al., 2024b). Notably, axonal integrity was regulated by O-GlcNAcylation on multiple proteins. For instance, heterogeneous nuclear ribonucleoprotein R interacted with OGT to co-regulate the O-GlcNAcylation of eukaryotic translation initiation factor 4G, thereby controlling axonal outgrowth in motor neurons and motor behavior in mice (Zare et al., 2024). In the peripheral nervous system, proper O-GlcNAcylation in Schwann cells maintained accurate protein localization within myelin sheaths, prevented axonal damage, and ameliorated sensory and motor nerve dysfunction (Kim et al., 2016). These studies have suggested that O-GlcNAcylation is indispensable for neuronal survival, as its specific knockout leads to somal degeneration and axonal withering, ultimately progressing to neuronal death (Su and Schwarz, 2017). Collectively, O-GlcNAcylation is implicated in multiple programmed cell death pathways.

Apoptosis, an orderly form of cell death controlled by genes, is crucial for maintaining homeostasis (Newton et al., 2024). The O-GlcNAcylation of protein kinase B (Akt) at Ser473 and Thr308 sites competitively inhibited its phosphorylation levels, thereby promoting neuronal apoptosis (Shi et al., 2015). During glutamate (Glu)-induced excitotoxicity, neuronal nitric oxide synthase (NOS) bound to OGT to catalyze the O-GlcNAcylation at the Thr119 and Ser283 sites of neuronal NOS, driving the formation of the complex between NOS and postsynaptic density (PSD)-95 to induce apoptosis (Chen et al., 2017). OGT knockdown reduced O-GlcNAcylation and rescued neuronal apoptosis and brain damage in mice (Zhao et al., 2022). In comparison, the upregulation of O-GlcNAcylation on the NOS adaptor protein reduced its interaction with neuronal NOS, thereby preventing neurons from Glu-induced apoptotic damage (Zhu et al., 2015). Furthermore, another study demonstrated that O-GlcNAcylation alleviated oxidative stress-injured apoptosis by increasing the protein expressions of signal transducer and activator of transcription 3 and forkhead box protein O1 (Zhang et al., 2024a).

Autophagy is an intracellular degradation pathway that facilitates the achievement of cellular metabolism and organelle renewal, but excessive autophagic activity may cause cell death (Zhang et al., 2024b). In rat primary cortical neurons, the upregulation of protein O-GlcNAcylation activated the mammalian target of rapamycin (mTOR) pathway, thereby excessively suppressing neuronal autophagy and impairing neuronal activity (Wani et al., 2017). Conversely, the downregulation of O-GlcNAcylation promoted autophagic flux by decreasing mTOR and increasing microtubule-associated protein 1 light chain 3-II (LC3-II) (Rahman et al., 2020). Moreover, O-GlcNAcylation also modulated the processing and trafficking of enzymatic proteins within the Golgi apparatus. O-GlcNAcylation of Golgi re-localizing and apparatus-specific phosphoprotein (GRASP55) impeded the fusion of lysosome-associated membrane glycoprotein 2 and LC3-II on autophagosomes, reducing autophagic fluxes (Zhang et al., 2018). Recent research has revealed that the function of O-GlcNAcylation in mitophagy is distinct from its role in overall cellular autophagy. O-GlcNAcylation activated mitochondrial autophagy protein phosphatase and tensin homologue-induced kinase 1 and LC3 levels to promote mitochondrial autophagy (Alghusen et al., 2024).

**Figure 4:**
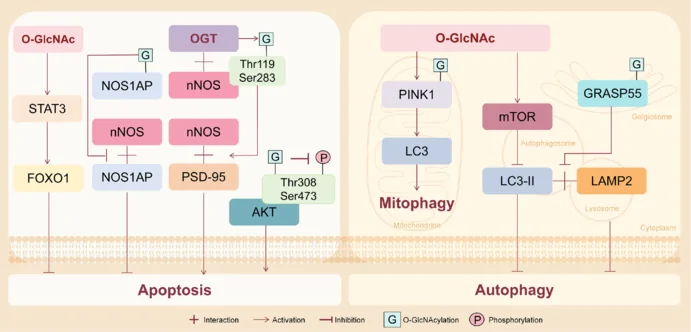
The role and representative proteins of O-GlcNAcylation in neuronal apoptosis and autophagy. Apoptosis: O-GlcNAcylation of nNOS and AKT promotes apoptosis. STAT3 and FOXO1 and O-GlcNAcylation of NOS1AP inhibit apoptosis. Autophagy: Overall O-GlcNAcylation and O-GlcNAcylated-GRASP55 inhibit cellular autophagy. Overall O-GlcNAcylation and O-GlcNAcylated-PINK1 promote mitochondrial autophagy. Created with Word Processing System Presentation (version 12.1.0.19302). AKT: Protein kinase B; FOXO1: forkhead box protein O1; GRASP55: Golgi re-localizing and apparatus-specific phosphoprotein; LAMP2: lysosome-associated membrane glycoprotein 2; LC3: microtubule-associated protein 1 light chain 3; mTOR: mammalian target of rapamycin; nNOS: neuronal nitric oxide synthase; NOS1AP: nitric oxide synthase adaptor protein; O-GlcNAc: N-acetylglucosamine; OGT: O-GlcNAc transferase; PINK1: phosphatase and tensin homologue-induced kinase 1; PSD-95: postsynaptic density-95; Ser: serine; STAT3: signal transducer and activator of transcription 3; Thr: threonine.

Neurons in neurodegenerative diseases exhibit a dying pattern, initiating with cytoskeletal disintegration and culminating in the fragmentation and loss of distal axons (Hilton et al., 2024). The protein O-GlcNAcylation maintains neuronal structures including axons and cytoskeletons, and ameliorates motor deficits in innervation. Despite occasional contradictory findings and potential adverse effects, it is evident that neuronal death mechanisms reliant on autophagy and apoptosis can be controlled by protein O-GlcNAcylation (**Figure 4**). Thus, sustaining O-GlcNAcylation homeostasis represents a promising therapeutic strategy to rescue neuronal injury and death.

### Signaling and communication of neurons

In the vast array of synapses in the brain, presynaptic membrane, synaptic cleft, and postsynaptic membrane specializations form a transcellular unit to facilitate effective information transfer between neurons (Biederer et al., 2017). Synaptic plasticity is a vital foundation for information processing, memory, and learning in the brain (Magee and Grienberger, 2020; Li and Jin, 2025; Liu et al., 2025). The O-GlcNAcylation of synaptophysin at Thr-87 regulated the size and release of the synaptic vesicle reserve pool, which was essential for maintaining synaptic plasticity (Skorobogatko et al., 2014). OGA mutant mice exhibited impaired phosphorylation of Glu α-amino-3-hydroxy-5-methyl-4-isoxazole propionic acid receptor (GluA) 1 subunit, triggering defects in hippocampal synaptic plasticity. Specifically, long-term potentiation and long-term depression were impaired, resulting in learning and memory damage (Yang et al., 2017). A novel O-GlcNAc-dependent long-term depression was regulated by the GluA2 subunit. This form of long-term depression was activated by the heightened O-GlcNAcylation, which suppressed pathological over-excitation (Stewart et al., 2017).

PSD is a protein-rich assembly located beneath the postsynaptic membrane, which concentrates a large number of excited and inhibited neurotransmitter receptors, involved in the signal transduction integration in the brain (Kaizuka and Takumi, 2024). OGT was accumulated in the PSD of excitatory synapses and promoted O-GlcNAcylation of various proteins during neuronal stimulation. However, the knockout of postsynaptic OGT resulted in a decreased level of the GluA2 and GluA3 subunits, a sharp reduction in the amount of excitatory presynaptic terminals, and a progressive immaturity of dendritic spines in neurons (Lagerlöf et al., 2017). Liquid-liquid phase separation of PSD-95 and synaptic Ras guanosine triphosphate hydrolases activating protein, two proteins expressed highly in PSD, was negatively regulated by O-GlcNAcylation, thereby affecting excitatory synaptic transmission (Lv et al., 2022).

Glu, as an excitatory neurotransmitter, is involved in neural signal transmission, synaptic plasticity, and neural network regulation (Reiner and Levitz, 2018). Under stress conditions in the brain, OGT was specifically upregulated in astrocytes, increasing the O-GlcNAcylation level of glutamate transporter-1. The change disrupted glutamatergic synaptic transmission, which exacerbated the symptoms of mental disorders (Fan et al., 2023). Gamma-aminobutyric acid (GABA) is the most important inhibitory neurotransmitter in the nervous system (Ngo and Vo, 2019). OGT-1, a homolog of OGT in *Caenorhabditis elegans* (*C. elegans*), was primarily expressed in the presynaptic terminals of GABAergic motor neurons (Giles et al., 2019). It was regulated by the insulin receptor dauer abnormally forming gene 2, which drove presynaptic assembly and protected synapse formation (Wu et al., 2023a). The dramatic increase in O-GlcNAc could inhibit GABAergic currents and GABA receptors in hippocampal neurons through postsynaptic mechanisms, thereby modulating the overall excitatory and inhibitory balance of neurons (Stewart et al., 2020; Phillips et al., 2024).

**Figure 5:**
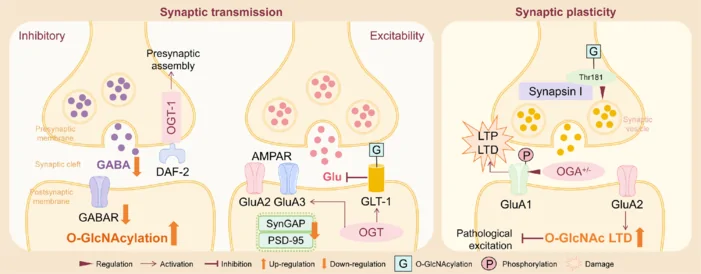
The role of O-GlcNAcylation in synaptic function and the representative receptors, transmitters, and proteins. Synaptic transmission: O-GlcNAcylation regulates inhibitory neurotransmission represented by GABA and excitatory neurotransmission represented by Glu. Synaptic plasticity: O-GlcNAcylation is involved in regulating LTP and LTD through AMPAR (GluA1/2). Created with Word Processing System Presentation (version 12.1.0.19302). AMPAR: α-Amino-3-hydroxy-5-methyl-4-isoxazole propionic acid receptor; DAF-2: dauer abnormally forming gene 2; GABA: gamma-aminobutyric acid; GABAR: GABA receptor; GLT-1: glutamate transporter-1; Glu: glutamate; GluA1/2/3: Glu α-amino-3-hydroxy-5-methyl-4-isoxazole propionic acid receptor 1/2/3; LTD: long-term depression; LTP: long-term potentiation; O-GlcNAc: N-acetylglucosamine; OGA: O-GlcNAcase; OGT: O-GlcNAc transferase; PSD-95: postsynaptic density-95; SynGAP: synaptic Ras guanosine triphosphate hydrolases activating protein; Thr: threonine.

Furthermore, O-GlcNAcylation is indispensable for regulating diverse sensory signaling pathways. OGT integrated nutritional and activity-related cues to modulate sugar responsiveness in gustatory neurons and sweet taste perception in *Drosophila* (Sung et al., 2023). In the hypothalamus, OGT and O-GlcNAcylation were highly abundant in Agouti-related protein neurons, where they regulated neuronal excitability via voltage-gated potassium channels to mediate fasting signals (Ruan et al., 2014). A reduction of protein O-GlcNAcylation in early embryogenesis led to decreased brain size and impaired olfactory learning in adult Drosophila, suggesting a potential mechanism for O-GlcNAc-related progressive intellectual disability (Zhang et al., 2023). In brief, O-GlcNAcylation serves as a signaling sensor that is abundantly expressed in the nervous system and essential for synaptic excitability, inhibition, and plasticity (**Figure 5**).

### Energy and metabolism of neurons

Mitochondria are the primary sites of cellular biooxidation and energy conversion, whose dysfunction is a hallmark of age-related neurodegenerative disorders (Park et al., 2021b). Neuronal activity is a glucose-intensive process, which can be compensated by promoting O-GlcNAcylation in mitochondria (Yu et al., 2024). The mitochondrial motor adaptor protein Milton has been identified as a substrate necessary for OGT, with its O-GlcNAcylation being regulated based on glucose availability, thereby influencing mitochondrial dynamics in neurons (Pekkurnaz et al., 2014). When glucose was lowered to critical levels, O-GlcNAc dysregulation induced mitochondrial dysfunction and cerebral glucose hypometabolism, resulting in Alzheimer’s disease-like changes in cortical neurons (Huang et al., 2023). Therefore, under conditions of glucose deprivation and reoxygenation stress, increasing O-GlcNAcylation can maintain mitochondrial homeostasis and improve bioenergy, thereby exerting neuroprotective effects (Xu et al., 2021a). This beneficial effect may be closely related to the regulation of the O-GlcNAc-dependent HBP. As reported, O-GlcNAcylation of hexokinase 1 enhanced its interaction with mitochondria, coordinating glycolysis and ATP production (Wang et al., 2024). Glutamine-fructose-6-phosphate transaminase enzyme activity was reduced in iron deficiency, which downregulated overall O-GlcNAcylation levels in neurons (Erber et al., 2021). In contrast, highly O-GlcNAcylated mitochondria counteracted glycosylation and reduced advanced glycation end product-mediated oxidative stress and autophagy, while improving the vitality of mitochondrial receptor neuronal (Park et al., 2023b).

O-GlcNAcylation manages cellular trophic status and coordinates the metabolism of damaged neurons to maximize regenerative efficiency (Taub et al., 2018). Beyond glucose metabolism, O-GlcNAcylation also influences other metabolic pathways. For example, the OGT-1 mutation resulted in significant transcriptional changes in genes involved in glycolysis, one-carbon metabolism, sulfur metabolism, and ATP metabolism. Among them, methionine, a one-carbon metabolism metabolite, was upregulated by OGT-1 to improve neuronal damage (Yadav et al., 2024). Furthermore, OGT knockout in adult mouse neurons induced short-term binge eating, weight gain, and peripheral insulin resistance (Dai et al., 2018). Acute deletion of OGT in αCaMKII-positive neurons within the hypothalamic paraventricular nucleus induced hyperphagia-driven obesity (Lagerlöf et al., 2016). Similarly, mice with ventromedial paraventricular nucleus neuron-specific OGT deficiency exhibited rapid weight gain, exacerbation of obesity, and decreased energy expenditure, indicating that O-GlcNAcylation underlined lipid metabolism regulation (Wang et al., 2022).

In summary, disruption of mitochondrial homeostasis is a pivotal pathological mechanism underlying the initiation and development of neurodegenerative diseases (Cisneros et al., 2022). By dynamically modulating the mitochondrial energy metabolic network, O-GlcNAcylation serves as a critical molecular nexus linking nutrient sensing to mitochondrial homeostasis, potentially exerting regulatory effects in delaying neuronal degeneration processes.

## Effects of O-GlcNAcylation in Alzheimer’s Disease

Alzheimer’s disease is a chronic neurological deterioration marked by progressive memory loss, cognitive dysfunction, and incapacitation in daily living (Twarowski and Herbet, 2023). Pathologically, Alzheimer’s disease manifests as atrophy of the cerebral cortex and hippocampus, accompanied by reduced synaptic connectivity between neurons (Pini et al., 2016; Griffiths and Grant, 2023). Hallmark neuropathological features include extracellular Aβ plaque deposition and intraneuronal neurofibrillary tangles formed by hyperphosphorylated tau protein (Chen et al., 2020a; Otero-Garcia et al., 2022). Compared to healthy controls, mature Alzheimer’s disease patients exhibit a substantial decline (47%) in whole protein O-GlcNAcylation levels in the frontal cortex (Frenkel-Pinter et al., 2017). Furthermore, diminished O-GlcNAcylation has been found in both Alzheimer’s disease *in vivo* models (3xTg Alzheimer’s disease transgenic mice) and *in vitro* models (SH-SY5Y cells simulating Alzheimer’s disease state) (Ding et al., 2018; Pinho et al., 2019). These pathological accumulations and their downstream learning and memory deficits are reversible by enhancing O-GlcNAcylation through improved glucose metabolism (Kim et al., 2024a). Multiple intervening factors have been shown to modulate Alzheimer’s disease status by O-GlcNAcylation (**Table 1**). Thus, the interplay and mechanistic relationships between O-GlcNAcylation and tau/Aβ pathologies deserve further investigation.

**Table 1:** Intervention conditions affecting AD through O-GlcNAcylation

Intervention conditions	Experimental subjects	O-GlcNAcylation	Pathological condition				Reference
			Aβ	Tau	Cognitive function	Others	
Repeated sleep deprivation	Repeated sleep deprivation zebrafish models	Decreased OGT and increased OGA	Increased O-GlcNAcylated APP and increased β-secretase activity		Impairment of learning and memory	Neuroinflammation, decreased GABA and Glu	Park et al., 2023a
Chronic rapid eye movement sleep deprivation	Chronic rapid eye movement sleep deprivation mouse models	Decreased levels of O-GlcNAcylation	Increased Aβ accumulation	Decreased O-GlcNAcylated tau and activated GSK3β	Dysfunction of long-term memory and fear memory	Neuroglia activation, ER stress, decreased dendritic spine density, increased PSD-95	Kim et al., 2024a
Repetitive hypoxia	Repetitive hypoxia zebrafish models	Decreased brain O-GlcNAcylation and increased OGA	Increased Aβ accumulation		Impairment of anxiety, learning, and memory	Brain inflammation	Park et al., 2021a
Oxidative stress	H_2_O_2_-injured SH-SY5Y cells	Overall O-GlcNAc increased rapidly, peaked at 2 h, and returned to baseline at 24 h		Decreased phosphorylated tau at Ser199 and PHF sites			Kátai et al., 2016
Low glucose	Pluripotent stem cells	O-GlcNAc level decreased after 6 h of low glucose treatment	Increased Aβ_1–42_ and Aβ_1–40_	Increased phosphorylated tau at Thr231, Thr181, Ser404, and Ser202		Loss of neurons and synapses, decreased mitochondrial function	Huang et al., 2023
Chronic hyperglycemia	KKAy mice, and AGE-treated HT22 cells	Decreased O-GlcNAcylation		Increased phosphorylated tau at Thr231, Thr212, Ser396, and Ser404	Impairment of learning and memory	Obesity, hyperinsulinemia, hyperglycemia	Huang et al., 2020
Metabolic syndrome	KKAy mice	Decreased O-GlcNAcylation		Increased phosphorylated tau in hippocampus-associated dorsal midbrain	Impairment of motor and cognitive function	Increased body weight, blood glucose, total cholesterol and triglycerides	Gupta et al., 2023
Fasting	Fasting Kunming mice	Decreased O-GlcNAcylation of tau		Tau hyperphosphorylation and neurogenic fiber degeneration			Li et al., 2006

AD: Alzheimer’s disease; AGE: advanced glycation end product; APP: amyloid precursor protein; Aβ: amyloid-beta; ER: endoplasmic reticulum; GABA: gamma-aminobutyric acid; Glu: glutamate; H_2_O_2_: hydrogen peroxide; PSD-95: postsynaptic density-95; GSK3β: glycogen synthase kinase 3 beta; O-GlcNAc: N-acetylglucosamine; OGA: O-GlcNAcase; OGT: O-GlcNAc transferase.

### O-GlcNAcylation and tau

**Figure 6:**
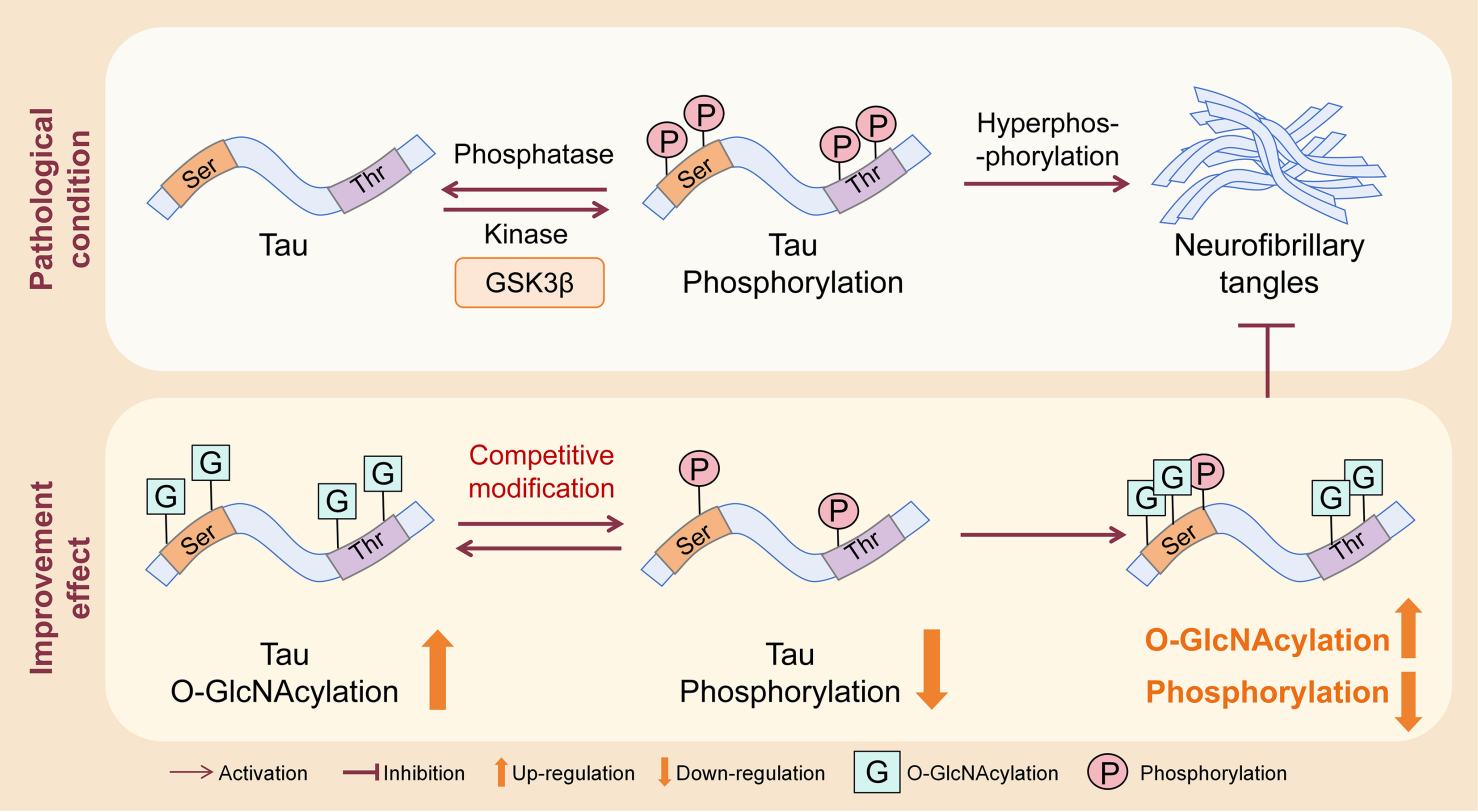
The role of O-GlcNAcylation in tau protein phosphorylation. In pathological states, kinase-mediated hyperphosphorylation of tau proteins forms neurotoxic Neurofibrillary tangles. However, O-GlcNAcylation, also located at Ser and Thr residues of tau protein, competitively inhibits tau phosphorylation, thereby reducing the accumulation of pathological neurofibrillary tangles. Created with Word Processing System Presentation (version 12.1.0.19302). GSK3β: Glycogen synthase kinase 3 beta; O-GlcNAc: N-acetylglucosamine; Ser: serine; Thr: threonine.

Tau proteins regulate, maintain, and stabilize microtubule assembly under physiological situations. In specific pathological states, tau is subject to post-translational modifications primarily via phosphorylation and glycosylation (Ahmad et al., 2020). In Alzheimer’s disease patient brains, tau O-GlcNAcylation is significantly decreased (Robertson et al., 2004), and non-hyperphosphorylated tau exhibits 4-fold higher O-GlcNAc content than hyperphosphorylated tau, demonstrating an inverse relationship between O-GlcNAcylation and phosphorylation (Liu et al., 2009). A similar relationship was identified in animal models. In the 3xTg Alzheimer’s disease mice, O-GlcNAcylation levels of tau protein were significantly reduced in the hippocampus, whereas no such change was observed in the frontal cortex or cerebellum (Gatta et al., 2016). In the tauopathy mice, the O-GlcNAcylation of tau did not alter the conformational characteristics of the monomeric protein, but it facilitated monomer dissolution, disrupted the homeostasis of fibrils or soluble aggregates, and ultimately reduced the accumulation of tau aggregates (Yuzwa et al., 2014). Therefore, the chronic disequilibrium between phosphorylation and O-GlcNAcylation of tau is regarded as one of the key features of Alzheimer’s disease (**Figure 6**).

Phosphorylation and O-GlcNAcylation of tau are predominantly located on Thr and Ser residues of the protein (Kátai et al., 2016), whose specific action sites have emerged as focal points in contemporary investigations. The immunoprecipitation-liquid chromatography-mass spectrometry method identified three O-GlcNAcylated tau sites in mice overexpressing tau, including Ser208, Ser191, and Ser184/Ser185 (Bijttebier et al., 2023). In individuals with mild cognitive impairment, the proportion of overall O-GlcNAcylation to tau phosphorylation at the Thr212 site in blood has been shown to decrease, along with memory impairment (Huang et al., 2019). In diabetic cognitively dysfunctional mice and advanced glycation end product-injured HT22 cells, O-GlcNAcylation expressions were reduced, and tau phosphorylation levels were increased at Thr231, Thr212, Ser404, and Ser396, but were reversed by overexpressing OGT (Huang et al., 2020). In contrast, inhibiting OGT resulted in a 1.5-fold elevation at Ser396 and a 2.0-fold elevation at Ser199 in tau phosphorylation, leading to increased tau aggregation (Lim et al., 2015). These pathological tau aggregates can be alleviated by inhibiting OGA specifically. In mice treated with pharmacological OGA inhibitors, O-GlcNAcylation expressions were upregulated, and tau phosphorylation at Ser404, Ser214, Ser409, Ser262/Ser356, Thr212, and Thr181 was reduced (Yu et al., 2012). Long-term treatment with an OGA inhibitor significantly improved tau O-GlcNAcylation and decreased the number of dystrophic neurons, preventing pathological tau formation but not altering the phosphorylation of non-pathological tau (Graham et al., 2014). In P301L tauopathy mice, OGA inhibition increased the overall O-GlcNAc levels in the brain and decreased both soluble and insoluble highly phosphorylated tau (Hastings et al., 2017; Rostgaard et al., 2023), accompanied by the improvement of motor deficits and survival time (Borghgraef et al., 2013).

O-GlcNAcylation is also responsible for modulating glycogen synthase kinase 3 beta (GSK3β), a prominent kinase for tau. GSK3β was significantly reduced following OGA knockout, thereby alleviating tau hyperphosphorylation (St Amand et al., 2018). Mechanistically, O-GlcNAcylation attenuates GSK3β-mediated phosphorylation at the PHF-1 site, slowing fibril assembly (Cantrelle et al., 2021). In addition, GSK3β itself could be highly O-GlcNAcylated at multiple sites within the N/C-terminal and kinase structural domains (El Hajjar et al., 2024). In conclusion, these findings have suggested that enhanced tau O-GlcNAcylation in Alzheimer’s disease reduces excessive phosphorylation, thereby decreasing the formation of neurofibrillary tangles. This effect is also associated with the downregulation of GSK3β kinase activity by O-GlcNAcylation.

### O-GlcNAcylation and amyloid-beta

Amyloid plaques formed by Aβ aggregation are considered an important hallmark of Alzheimer’s disease. These plaques can interfere with normal signaling between nerve cells and generate oxidative stress, leading to cell damage and death (Karisetty et al., 2020). Excessive glucose accumulation impaired neurotransmission and increased the production of toxic Aβ oligomers (Bukke et al., 2020). O-GlcNAcylation, directly related to glucose metabolism, has been shown to reduce Aβ toxicity (Ahmad, 2022). However, the loss of OGT specifically in the forebrain of adult mice caused downregulation of O-GlcNAc levels, resulting in increased Aβ peptide production and memory impairment (Wang et al., 2016). Restoring O-GlcNAcylation function inhibited astrocyte activation, reduced inflammation, and relieved Aβ plaque aggregation in Alzheimer’s disease mice (Dong et al., 2023). Additionally, the IXI domain of small heat shock proteins bound to O-GlcNAc interfered with the Aβ_1–42_ production, exerting anti-amyloid activity (Balana et al., 2021). Notably, the regulatory effect of O-GlcNAcylation and Aβ is bidirectional, that is, Aβ can affect O-GlcNAcylation of specific proteins. For instance, Aβ promoted O-GlcNAcylation of c-Fos, which exacerbated neuronal death (Choi et al., 2019), and regulated mitochondrial fission by promoting the O-GlcNAcylation of Drp1 (Park et al., 2021c). Moreover, Aβ directly interacted with ATP synthase and reduced the O-GlcNAcylation of the ATP synthase subunit α, thus decreasing ATPase activity and ATP yield (Cha et al., 2015).

**Figure 7:**
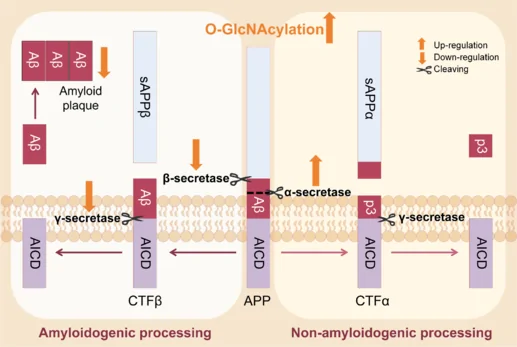
The effect of O-GlcNAcylation on the Aβ production pathway. O-GlcNAcylation increases α-secretase production and promotes the non-amyloidogenic pathway. Meanwhile, O-GlcNAcylation decreases the production of β-secretase and γ-secretase to inhibit the amyloidogenic pathway and Aβ aggregation. Created with Word Processing System Presentation (version 12.1.0.19302). AICD: APP intracellular domain; APP: amyloid precursor protein; Aβ: amyloid-beta protein; CTFα/β: C-terminal fragment alpha/beta; O-GlcNAc: N-acetylglucosamine; sAPPα/β: soluble APP alpha/beta; α/β/γ-secretase: alpha/beta/gamma-secretase.

The mechanism by which O-GlcNAcylation modulates Aβ peptide toxicity primarily involves regulating the amyloidogenic pathway (**Figure 7**). APP is the precursor for Aβ generation. The portion of APP at the plasma membrane cleaved by α-secretase and γ-secretase does not form Aβ fragments, whereas the portion cleaved by β-secretase and γ-secretase forms Aβ plaques (Cho et al., 2022). The expression of O-GlcNAcylation on APP has a significant role in Aβ production. On the one hand, upregulation of O-GlcNAcylation promoted α-secretase processing, resulting in an elevation of the neuroprotective soluble APP alpha fragment and a decline in Aβ secretion, thus enhancing the non-amyloidogenic pathway (Jacobsen and Iverfeldt, 2011). On the other hand, the increase in O-GlcNAcylation reduced the activity of β-secretase and γ-secretase, decreasing Aβ generation and attenuating the amyloidogenic pathway (Kim et al., 2013; Park et al., 2023a). Furthermore, O-GlcNAcylation at the Thr576 residue of APP enhanced its transport rate to the plasma membrane, thereby reducing Aβ generation (Chun et al., 2017). Another study also demonstrated that inhibiting OGA increased the transport rate of APP from the trans-Golgi network to the plasma membrane and decreased APP endocytosis, thereby enhancing non-amyloid production and processing (Chun et al., 2015). These findings have suggested that modulating O-GlcNAcylation to regulate Aβ production becomes a potential treatment option for Alzheimer’s disease.

## Effects of O-GlcNAcylation in Parkinson’s Disease

Parkinson’s disease, the second most common neurodegenerative disorder of the central nervous system (CNS) after Alzheimer’s disease (Bloem et al., 2021; Salahuddin et al., 2021), is clinically marked by core motor symptoms including resting tremor, bradykinesia, and rigidity (Burtscher et al., 2024). Non-motor symptoms such as hyposmia, sleep disturbances, autonomic dysfunction, and cognitive decline frequently accompany disease progression (Leite Silva et al., 2023). Pathologically, Parkinson’s disease is defined by the formation of Lewy bodies formed by abnormal aggregation of α-syn and the gradual loss of dopaminergic neurons in the substantia nigra of the midbrain (Kim et al., 2025; Yan et al., 2025). Recent studies showed the critical role of O-GlcNAcylation in Parkinson’s disease pathogenesis through its regulation of dopamine metabolism and α-syn aggregation (Hu et al., 2024; Kim et al., 2024b).

### O-GlcNAcylation and dopaminergic neurons

In Parkinson’s disease, the degeneration of dopaminergic neurons in the substantia nigra leads to reduced dopamine synthesis and subsequent motor dysfunction (Furukawa et al., 2022). O-GlcNAcylation has multifaceted regulatory effects on dopaminergic neurons across molecular, synaptic, cellular, and behavioral aspects. For example, pharmacological or genetic increase in O-GlcNAcylation attenuated neurodegeneration, synaptic impairment, and motor defect in Parkinson’s disease animal models (Lee et al., 2020). In Parkinson’s disease-like mice and Neuro-2a cells impaired by 6-hydroxydopamine, the overall level of O-GlcNAcylation was dose-dependently down-regulated, accompanied by elevated OGA expression. However, restoring O-GlcNAcylation via glucosamine (GlcN) supplementation or OGT overexpression suppressed neuronal death, alleviated neuroinflammation and mitochondrial dysfunction, and ameliorated motor impairments (Kim et al., 2024b). Notably, O-GlcNAcylation exhibited bidirectional regulatory properties. While inhibiting OGA in the ventral tegmental area elevated O-GlcNAc levels in dopaminergic neurons, it impaired natural reward responsiveness and motor learning in mice, indicating that excessive O-GlcNAcylation may paradoxically harm dopaminergic function (Shao et al., 2022).

Tyrosine hydroxylase (TH), the rate-limiting enzyme in dopamine biosynthesis, catalyzes the conversion of tyrosine to L-3,4-dihydroxyphenylalanine, a direct precursor of dopamine (Costa and Schoenbaum, 2022; Kolacheva et al., 2022). Therefore, the phosphorylation and activation of TH can effectively promote the increase of dopamine synthesis (Bueno-Carrasco et al., 2022; Douma et al., 2024). However, O-GlcNAcylation at Ser and Thr residues on TH competitively inhibited its phosphorylation, thereby reducing dopamine production (Bork et al., 2008). Conversely, decreased O-GlcNAcylation in dopaminergic neurons upregulated phosphorylation at Ser40 of TH, significantly increasing intracellular L-3,4-dihydroxyphenylalanine levels (da Costa Rodrigues et al., 2025). These findings have demonstrated that TH activity is antagonistically regulated by O-GlcNAcylation and phosphorylation, dynamically modulating L-3,4-dihydroxyphenylalanine and dopamine metabolic homeostasis.

### O-GlcNAcylation and alpha-synuclein

α-syn, the core pathological protein in Parkinson’s disease, forms Lewy bodies and damages neurons through abnormal aggregation (Leak et al., 2024). Current investigations have revealed a pivotal nexus between O-GlcNAcylation processes and α-syn aggregation mechanisms (Marotta et al., 2012). Comparing the effects of O-GlcNAc with other similar monosaccharides such as glucose, N-acetylgalactosamine, or mannose on the formation of α-syn amyloid protein, it was found that O-GlcNAc was the only monosaccharide that strongly inhibited α-syn amyloid protein accumulation by different characterization techniques (Galesic et al., 2021). All-atom molecular dynamics simulations further indicated that O-GlcNAcylation disrupted hydrogen bond formation between α-syn monomers, sterically hindering oligomerization through spatial effects (Wu et al., 2020). Additionally, O-GlcNAcylated α-syn generated a non-pathogenic amyloid strain *in vivo*, altering the interactome of α-syn preformed fibrils (PFFs) and reducing seeding activity, thereby mitigating Parkinson’s disease progression (Balana et al., 2024). O-GlcNAcylation also regulated α-syn PFFs formation and internalization. OGA inhibition reduced α-syn PFFs uptake in a time- and concentration-dependent manner in multiple cell lines, including murine primary cortical neurons, whereas OGT inhibition enhanced α-syn PFFs internalization (Tavassoly et al., 2021). Paradoxically, a study found increased levels of the protein O-GlcNAcylation in lysates from the temporal cortex of Parkinson’s disease patients. Excessive O-GlcNAcylation activated mTOR in rat primary cortical neurons, reduced autophagic flux, and increased α-syn deposition, which was detrimental to neurons (Wani et al., 2017).

Current research has pinpointed specific O-GlcNAcylation sites on α-syn. This 140-amino acid protein comprises three functional domains: the N-terminal repeat domain (1–60 residues) (McGlinchey et al., 2021), central hydrophobic domain (61–95 residues), and C-terminal domain (96–140 residues) (Farzadfard et al., 2022). The central domain contains the non-Aβ-component (NAC), a critical driver of pathological self-aggregation (Kumar et al., 2022). Site-specific O-GlcNAc functionalization within the NAC domain altered peptide self-assembly propensity and toxicity (Ryan et al., 2020). Proteomic analyses in mice and humans identified at least nine O-GlcNAc-modified residues (Thr33, Thr44, Thr54, Thr59, Thr64, Thr72, Thr75, Thr81, and Ser87) on α-syn, with multiple sites (Thr72, and Ser87) localized to the NAC or adjacent regions, directly modulating aggregation dynamics (Levine et al., 2019).

Both Thr72 and Ser87 O-GlcNAcylation suppressed α-syn aggregation, potentially by inhibiting calpain-mediated α-syn proteolysis, thereby influencing protein degradation and aggregation (Levine et al., 2017). Single-molecule dynamics of O-GlcNAcylated α-syn (Thr72, and Ser87) revealed distinct conformational effects. O-GlcNAcylated α-syn at Thr72 made the protein less compact and more diffusive, while O-GlcNAcylated α-syn at Ser87 stabilized the protein conformation with reduced diffusivity (Gamage et al., 2024). O-GlcNAcylated α-syn at Thr72 inhibited α-syn stoichiometry without affecting its membrane-binding or bending properties (Marotta et al., 2015). O-GlcNAcylated α-syn at Ser87 also inhibited α-syn aggregation, but to a lesser extent than the same modification at Thr72, and did not affect the membrane-bound properties of α-syn (Lewis et al., 2017). Cryo-electron microscopy demonstrated that phosphorylated and O-GlcNAcylated α-syn at Ser87 formed distinct fibril architectures with decreased neurotoxicity and propagation activity compared to unmodified fibrils (Hu et al., 2024). To resolve the heterogeneity of α-syn O-GlcNAcylation, a novel synergistic technique of trapped ion mobility and mass spectrometry enabled high-coverage (80%) identification of glycoforms at Thr72, Thr75, Thr81, and Ser87, offering the potential for large-scale O-GlcNAcylation glycoform screening (Miller et al., 2023).

**Figure 8:**
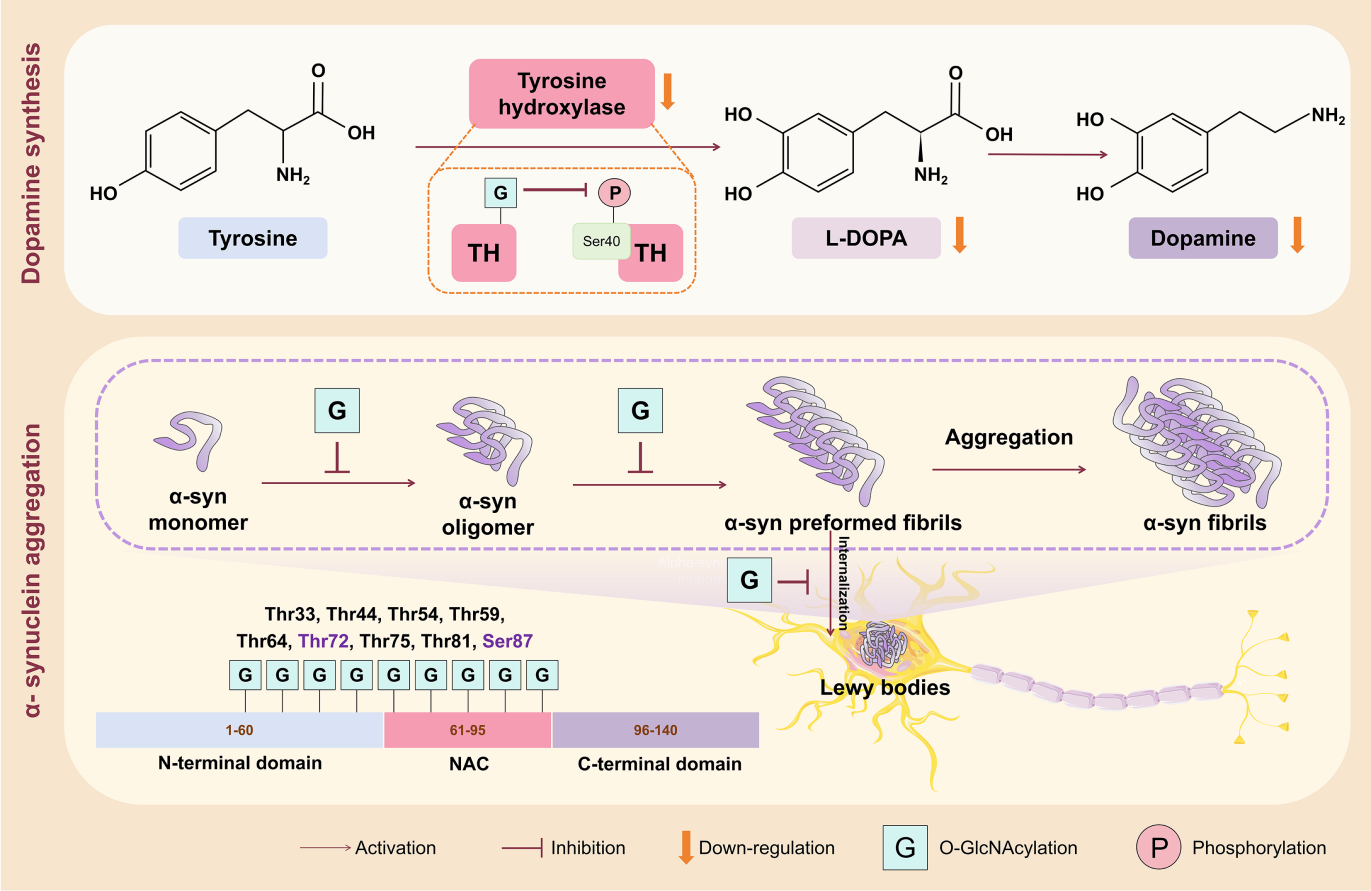
Regulatory effects of O-GlcNAcylation on dopamin and α-syn in PD. O-GlcNAcylation exerts dual regulatory effects. In dopaminergic biosynthesis, it suppresses phosphorylation and activation of TH, thereby reducing L-DOPA and dopamine production. Regarding α-synuclein pathology, O-GlcNAcylation demonstrates neuroprotective potential by inhibiting oligomerization, fibrillization of α-syn, and internalization of preformed fibrils. Created with Word Processing System Presentation (version 12.1.0.19302). L-DOPA: L-3,4-dihydroxyphenylalanine; NAC: non-amyloid beta-component; O-GlcNAc: N-acetylglucosamine; PD: Parkinson’s disease; Ser: serine; TH: tyrosine hydroxylase; Thr: threonine; α-syn: alpha-synuclein.

In summary, O-GlcNAcylation exerts multidimensional regulatory roles in Parkinson’s disease pathogenesis (**Figure 8**). In dopaminergic neurons, it dynamically balances TH phosphorylation to modulate dopamine synthesis while sustaining neuronal survival and function. In α-syn pathology, site-specific O-GlcNAcylation (Thr72 and Ser87) mitigates neurotoxicity through conformational stabilization, aggregation suppression, and enhanced degradation pathways.

## Effects of O-GlcNAcylation in Amyotrophic Lateral Sclerosis

Amyotrophic lateral sclerosis, a chronic progressive neurodegenerative disorder, is pathologically marked by the gradual loss of both upper and lower motor neurons (Feldman et al., 2022; Wlaschin et al., 2023). Clinically, patients commonly present with core symptoms such as progressive skeletal muscle weakness, atrophy, and bulbar paralysis, which progressively worsen over time (Rosina et al., 2025). Recent studies have revealed that aberrant O-GlcNAcylation plays a critical regulatory role in amyotrophic lateral sclerosis pathogenesis, particularly in motor neuron degeneration and pathological protein aggregation. A quantitative chemoproteomic analysis investigated O-GlcNAcylation dynamics in the lumbar spinal cords of amyotrophic lateral sclerosis model mice (SOD1-G93A) and non-transgenic control mice. Among 568 high-confidence O-GlcNAcylation sites identified in both groups, 226 sites dynamically regulated were predominantly localized to proteins associated with neuronal function and structural integrity (Hao et al., 2025).

The accumulation of mutant superoxide dismutase 1 (SOD1) protein in motor neurons contributes to amyotrophic lateral sclerosis pathogenesis (Peggion et al., 2022). In transgenic animal models overexpressing mutant SOD1, reduced O-GlcNAc immunoreactivity in spinal motor neurons suggested a strong correlation between amyotrophic lateral sclerosis progression and O-GlcNAcylation dysregulation (Shan et al., 2012). Furthermore, mice deficient in the oxidative stress sensor glutathione peroxidase 7 (GPx7) exhibited amyotrophic lateral sclerosis-like phenotypes, including motor neuron loss, muscle denervation, and paralysis. Mechanistically, GPx7 deficiency impaired O-GlcNAcylation, leading to age-dependent oxidative stress damage and accelerated motor neuron degeneration (Hsieh et al., 2019). These findings collectively demonstrated that the oxidative stress-induced decline in O-GlcNAcylation played a pivotal role in motor neuron injury during amyotrophic lateral sclerosis progression.

Abnormal aggregation and mislocalization of TDP-43 represent hallmark pathological features of amyotrophic lateral sclerosis (Mitra et al., 2025; Omar et al., 2025). Pathogenic TDP-43 disrupted RNA metabolism, proteostasis, and mitochondrial function, ultimately triggering neuronal death (Dang et al., 2025). OGT-mediated TDP-43 O-GlcNAcylation suppressed its aggregation and hyperphosphorylation while enhancing its splicing activity. This modification ameliorated motor deficits in both larval and adult models and extended adult lifespan (Zhao et al., 2021). These studies highlighted the therapeutic potential of targeting protein modifications such as TDP-43 O-GlcNAcylation for amyotrophic lateral sclerosis intervention.

In amyotrophic lateral sclerosis, abnormal aggregation and accumulation of neurofilament proteins are recognized as critical contributors to neuronal injury (van Asperen et al., 2024). Neurofilaments, key components of the neuronal cytoskeleton (Marcus, 2022), are composed of neurofilament light, medium, and heavy chain (NF-L, NF-M, and NF-H) proteins. Their aberrant aggregation disrupts axonal transport and compromises neuronal structural integrity (Yuan and Nixon, 2021). NF-L, as the scaffold protein of neurofilaments, provides structural support and stability to neurons (Milo et al., 2020). O-GlcNAcylated NF-L is essential for normal organelle transport in primary neurons. Elevated O-GlcNAcylation at five NF-L residues (Thr21, Ser27, Ser34, Ser48, and Ser431) maintained NF-L in a low-oligomeric state, reduced the prevalence of full-length filaments, and modulated both assembly and pathological NF aggregation (Huynh et al., 2023). The phosphorylation regulation of NF-M is closely associated with neurofilament aggregation and disassembly processes, influencing their axonal distribution (Lefebvre-Omar et al., 2023). In spinal cord tissues of amyotrophic lateral sclerosis transgenic rat models, O-GlcNAcylation levels in the tail domain of NF-M were reduced, while total phosphorylation was increased (Lüdemann et al., 2005). This evidence suggested that O-GlcNAcylated NF-M was intricately and dynamically intertwined with phosphorylation cascades. NF-H, the largest neurofilament subunit, harbors mutations in its tail and rod domains that elevate amyotrophic lateral sclerosis risk (Marriott et al., 2024). Under glucose deprivation, the solubility of NF-H increased in an O-GlcNAc-dependent manner, indicating that O-GlcNAcylated NF-H modulated its disassembly from filaments (Cheung and Hart, 2008).

**Figure 9:**
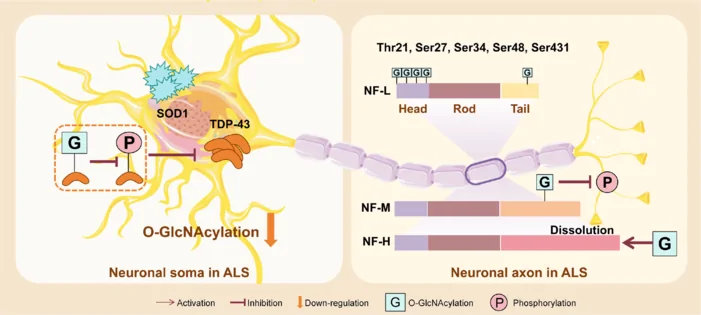
The effect of O-GlcNAcylation on neurons in ALS. ALS-affected neurons exhibit characteristic pathological accumulation of SOD1 and TDP-43 proteins concurrent with O-GlcNAcylation downregulation. Notably, O-GlcNAcylation of TDP-43 attenuates its phosphorylation while suppressing aberrant aggregation. Furthermore, O-GlcNAcylation regulates axonal neurofilament dynamics through three distinct mechanisms: direct modification of NF-L subunits, inhibition of NF-M phosphorylation, and stabilization of NF-H solubility. Created with Word Processing System Presentation (version 12.1.0.19302). ALS: Amyotrophic lateral sclerosis; NF-L/M/H: neurofilament-light/medium/heavy chain; O-GlcNAc: N-acetylglucosamine; SOD1: superoxide dismutase 1; TDP-43: transactive response DNA-binding protein 43.

Collectively, these studies elucidate the momentous role of O-GlcNAcylation in multi-level amyotrophic lateral sclerosis pathogenesis, spanning oxidative stress regulation, proteostasis maintenance, and cytoskeletal dynamics (**Figure 9**). Notably, O-GlcNAcylation suppresses hyperphosphorylation of TDP-43 and NF-M proteins, mitigates their abnormal aggregation, and alleviates motor neuron injury.

## Effects of O-GlcNAcylation in Huntington’s Disease

Huntington’s disease is an inherited neurodegenerative disorder characterized by midlife onset, choreiform movements, and progressive loss of speech, mobility, cognition, and swallowing abilities (Hu et al., 2025). Huntington’s disease is caused by an autosomal dominant mutation involving abnormal expansion of CAG trinucleotide repeats in the huntingtin (*HTT*) gene on chromosome 4, leading to misfolding and accumulation of mutant huntingtin protein (mHTT), which exerts neurotoxic effects (Tong et al., 2024).

Emerging evidence suggested that O-GlcNAcylation exerted complex and multifaceted roles in Huntington’s disease pathogenesis, potentially mediating both protective mechanisms and pathological exacerbation under specific conditions. In Huntington’s disease, aberrant mHTT aggregation directly disrupted nucleocytoplasmic transport via impairment of nuclear pore complexes. O-GlcNAcylation was implicated in maintaining nucleoporin levels within nuclear pore complexes (Zhu et al., 2016). Notably, NUP62, a core nucleoporin, was an important O-GlcNAcylation substrate. Reduced or dysregulated O-GlcNAcylation levels correlated with NUP62 mislocalization in postmortem Huntington’s disease brains, Huntington’s disease mouse models, and primary cortical neurons. However, elevating O-GlcNAcylation levels via OGA inhibitors restored nuclear pore stability, rescued nucleocytoplasmic transport deficits, and mitigated neuronal death (Grima et al., 2017). This protective effect appeared cell type- and disease stage-dependent. For instance, in Huntington’s disease *Drosophila* models and mHTT-expressing neuroblastoma cell lines, reducing O-GlcNAcylation enhanced autophagic flux, thereby reducing toxic mHTT aggregates (Kumar et al., 2014). Similarly, a *C. elegans* study demonstrated that O-GlcNAcylation accumulation significantly increased toxic protein expression in amphid single cilium type H neurons, exacerbating cell death in Huntington’s disease models (Wang et al., 2012).

Current research on O-GlcNAcylation in Huntington’s disease remains nascent, with unresolved questions regarding substrate specificity, mechanistic details, and conflicting findings that underscore the complexity of its roles. Future studies should prioritize elucidating the precise impact of O-GlcNAcylation on mHTT aggregation dynamics to unravel its potential bidirectional regulatory mechanisms in Huntington’s disease progression.

## Effects of O-GlcNAcylation in Aging

Aging, characterized by progressive functional decline in organisms over time, represents a major risk factor for neurodegenerative diseases (Culig et al., 2022). With advancing age, the nervous system suffers gradual damage and neuronal repair decreases, leading to the onset of neurodegenerative disorders marked by reduced physiological resilience, heightened disease susceptibility, and impaired homeostatic maintenance (Syed et al., 2024). During aging, the dynamic equilibrium of O-GlcNAcylation is disrupted, correlating with neurodegenerative pathologies and metabolic dysregulation (Banerjee et al., 2016). The O-GlcNAc cycle was found to critically influence processes from embryonic development to the maintenance of genome stability in aging (Yang et al., 2012).

O-GlcNAcylation exerted multi-dimensional regulatory effects on aging and longevity by modulating key protein functions. For instance, O-GlcNAcylated STAT1 at Thr699 blocked its β-hydroxybutyrylation at Lys592, enhancing type I interferon-mediated antiviral defenses in aged individuals (Zuo et al., 2025). Adult Drosophila mutants with OGA knockout exhibited increased body size but shortened lifespan. Proteomic analysis of these flies identified O-GlcNAcylated proteins, including host cell factor, SIN3 transcription regulator family member A, and forkhead box protein O, suggesting O-GlcNAcylation involvement in growth and longevity-associated pathways (Akan et al., 2021). In *C. elegans*, the age-dependent decline in OGT-1 expression reduced overall O-GlcNAcylation levels and shortened lifespan (Su et al., 2020). A further *C. elegans* study demonstrated that OGT-1 enhanced the antioxidant and pro-longevity activity of skinhead-1 via O-GlcNAcylation at Ser470 and Thr493, highlighting O-GlcNAcylation as a potential therapeutic target for aging interventions (Li et al., 2017).

Age-related decline in energy metabolism efficiency, impaired cellular energy supply, and accelerated senescence (Amorim et al., 2022). Mitochondria, as the cellular powerhouses, accumulated dysfunction with age and were closely linked to neurodegenerative pathologies (Zong et al., 2024). O-GlcNAcylation regulated the mitochondrial integrated stress response by modulating the activating transcription factor 4 (Alghusen et al., 2023). Aging also serves as a nexus for multiple chronic metabolic disorders (Spinelli et al., 2023). In aged livers, carbamoyl phosphate synthetase 1 underwent extensive O-GlcNAcylation. Hyperglycemia stimulated carbamoyl phosphate synthetase 1 O-GlcNAcylation and suppressed its activity, implicating that O-GlcNAcylation played a role in nutrient-sensing pathways during aging (Wu et al., 2022). Aging mouse models with metabolic syndrome exhibited sensory-motor and cognitive deficits accompanied by elevated phosphorylated tau and reduced O-GlcNAcylated protein levels, suggesting that diminished O-GlcNAc signaling led to metabolic dysregulation-induced aging cognitive dysfunction (Gupta et al., 2023). Moderate dietary restriction is a proven strategy to delay aging and improve metabolic health (Davis et al., 2016). For example, caloric restriction elevated O-GlcNAc and OGT levels, suppressed calcium/calmodulin-dependent protein kinase II, lipocalin-2, and phosphorylated tau expression, thereby ameliorating learning defects in obese mice (Jeon et al., 2016).

Aging-related neurological decline and cognitive impairment were tightly linked to spatiotemporal dynamics of O-GlcNAcylation. In the aging hippocampus, reduced O-GlcNAcylation in NSCs was correlated with diminished neurogenesis, increased gliogenesis, and impaired cognition (White et al., 2020). Intriguingly, enhancing O-GlcNAcylation rejuvenated cellular and cognitive functions in aged brains (Wang et al., 2021). Elevating brain protein O-GlcNAcylation improved motor deficits and extended survival in aged tau^P301L^ mice (Borghgraef et al., 2013). Overexpression of OGT in hippocampal neurons of young mice enhanced associative fear memory, while similar manipulation in aged neurons partially reversed deficits in fear and spatial memory, positioning O-GlcNAcylation as a critical mediator of cognitive rejuvenation (Wheatley et al., 2019).

By modifying specific proteins, regulating metabolic function, and alleviating cognitive deficits, O-GlcNAcylation plays a pivotal role in counteracting aging. Thus, deciphering the spatiotemporal heterogeneity of O-GlcNAcylation may unveil novel strategies to delay cognitive decline and extend health span.

## Effects of O-GlcNAcylation in Other Neurodegenerative Disorders

Machado-Joseph disease, also termed spinocerebellar ataxia type 3, is a common autosomal dominant neurodegenerative disorder (Costa, 2020). Elevated OGT levels and increased O-GlcNAc have been observed in Machado-Joseph disease cellular and animal models, suggesting a regulatory role of O-GlcNAc in Machado-Joseph disease pathogenesis (Pereira Sena et al., 2021). Clinically, Machado-Joseph disease manifests as cerebellar ataxia, pyramidal/extrapyramidal symptoms, progressive external ophthalmoplegia, distal muscle atrophy, facial fasciculations, and exophthalmos (Vasconcelos-Ferreira et al., 2022). The most common etiology involves mutations in the ataxin-3 gene, which trigger the expansion of the polyglutamine (polyQ) domain in the encoded protein. This aberrant polyQ extension confers a propensity for pathological aggregation, while misfolded ataxin-3 ultimately induces neuronal dysfunction and death (Potapenko et al., 2024). Notably, OGT directly interacted with ataxin-3 as a substrate. Reducing OGT levels or activity was potently associated with reduced ataxin-3 aggregation, improved protein clearance and cell viability, and attenuation of dyskinesias (Pereira Sena et al., 2021).

Giant axonal neuropathy is a rare inherited neurodegenerative disorder characterized by childhood-onset chronic progressive distal polyneuropathy, intellectual decline, curly hair, and skeletal muscle abnormalities (Bharucha-Goebel et al., 2021). Its pathology is linked to loss-of-function mutations in gigaxonin, an E3 ubiquitin ligase adaptor protein, and subsequent microtubule system disruption (Gangfuß et al., 2024). Gigaxonin maintains cytoskeletal homeostasis by regulating ubiquitination-dependent degradation of intermediate filament components such as vimentin and neurofilaments (Renganathan et al., 2023). Chen et al. (2020b) revealed that O-GlcNAcylation of gigaxonin facilitated its optimal binding to intermediate filament proteins, promoting their polyubiquitination and proteasomal degradation. Nutrient deprivation reduced O-GlcNAcylated gigaxonin at Ser272 and Thr277 residues, impairing gigaxonin-intermediate filament interactions, inducing gigaxonin accumulation, and exacerbating giant axonal neuropathy progression.

Multiple sclerosis is an autoimmune demyelinating and neurodegenerative disorder of the CNS (Marcus, 2022). Clinical features include recurrent neurological dysfunction, such as motor, sensory, visual, and cognitive impairments (Oh et al., 2018). The pathological mechanisms mainly involve abnormal responses of the immune system (Jelcic et al., 2025). Among them, T helper 17 (Th17) cells are considered to be one of the key pathological factors in the occurrence and development of multiple sclerosis (Mangani et al., 2024). Th17 cells infiltrated the CNS by crossing the blood-brain barrier, inducing inflammatory demyelination and neuronal damage (Peng et al., 2022). Mechanistically, Th17 cell differentiation was tightly regulated by the microRNA-15b/O-GlcNAc signaling axis. microRNA-15b suppressed OGT translation by binding to its mRNA 3’UTR, thereby reducing O-GlcNAcylation of critical transcription factors like NF-κB. Ultimately, overexpression of microRNA-15b inhibited Th17 differentiation and attenuated autoimmune encephalomyelitis (Liu et al., 2017a).

In a word, O-GlcNAcylation exerts neuroprotective effects in Machado-Joseph disease by enhancing the clearance of pathogenic ataxin-3 aggregates. In giant axonal neuropathy, O-GlcNAcylation regulates cytoskeletal protein ubiquitination to maintain axonal integrity. In multiple sclerosis, O-GlcNAcylation influences Th17 cell differentiation by regulating immune metabolism. These findings underscore the multidimensional regulatory roles of O-GlcNAcylation across diverse neurodegenerative diseases, highlighting their potential as therapeutic targets.

## O-GlcNAcylation Regulatory Strategies for Neurodegenerative Disease Therapy

### Hexosamine biosynthetic pathway modulators

HBP, the central regulatory hub of O-GlcNAcylation, utilizes its key metabolite GlcN to activate glutamine fructose-6-phosphate amidotransferase, thereby catalyzing the conversion of glucose to UDP-GlcNAc and significantly elevating intracellular O-GlcNAcylation levels (Parker et al., 2003). GlcN-mediated O-GlcNAc signaling modulates diverse pathological processes, including vascular inflammation (Xing et al., 2008, 2011; Hilgers et al., 2012), viral transcription (Jochmann et al., 2009), tumorigenesis (Kwak et al., 2010; Yu et al., 2019), embryonic migration/proliferation (Jeon et al., 2013), oxidative stress (Chen et al., 2015), and energy metabolism (Hwang et al., 2017; Park et al., 2020). Notably, GlcN exerts neuroprotective effects against neurological damage. In a rat middle cerebral artery occlusion model, intraperitoneal GlcN administration reduced infarct volume, alleviated motor deficits, and improved neurological outcomes via O-GlcNAcylation modulation (Hwang et al., 2010). In animal models of sleep deprivation and repetitive hypoxic exposure, GlcN supplementation restored cerebral O-GlcNAcylation, mitigating neurodegenerative pathologies such as neuroinflammation, Aβ and phosphorylated tau accumulation, and learning-memory impairments (Kim et al., 2021, 2024a; Park et al., 2021a, 2023a). Furthermore, GlcN attenuated 6-hydroxydopamine-induced Parkinson’s disease pathology by enhancing O-GlcNAcylation, thereby restoring dopaminergic neuronal function (Kim et al., 2024b). Although these studies highlighted GlcN’s potential in inflammation regulation, glucose metabolism, and neurodegenerative disorders, robust clinical evidence remained limited. Currently, GlcN is primarily utilized as a dietary supplement without formal therapeutic approval (DE LA Rosa et al., 2022).

### O-GlcNAc transferase inhibitors

Early research on OGT inhibitors focused on substrate analogs. Alloxan, the first reported OGT inhibitor, suppressed enzymatic activity by occupying the UDP-binding pocket, but its poor stability and low selectivity limited therapeutic utility (Konrad et al., 2002). Subsequently, donor substrate UDP-GlcNAc analogs evolved into potential OGT inhibitor scaffolds. UDP-C-GlcNAc, UDP-S-GlcNAc, and C-UDP thus emerged, but were limited by poor cell permeability and difficulty to act *in vivo* (Dorfmueller et al., 2011). In contrast, acetyl-5-thio-N-acetylglucosamine effectively penetrated cells and inhibited OGT activity both *in vitro* and *in vivo* (Gloster et al., 2011). Ac-5SGlcNAc drastically reduced whole UDP-GlcNAc levels and accelerated neuronal differentiation (Andres et al., 2017). Additionally, the OGT metabolic precursor inhibitor 2-deoxy-2-N-hexanamide-5-thio-d-glucopyranoside (5SGlcNHex) induced dose- and time-dependent reductions in O-GlcNAc levels across multiple tissues (Liu et al., 2018). For instance, 5SGlcNHex treatment increased α-syn PFFs uptake, accelerating the propagation of Parkinson’s disease-related pathology (Tavassoly et al., 2021). ES1, the first covalent inhibitor targeting OGT, irreversibly inhibited its activity in a time-dependent manner *in vitro* (Worth et al., 2019). Host cell factor-1, an OGT proteolytic substrate, generated a derived peptide that bound with high affinity to a conserved asparagine residue in the OGT TPR domain, effectively inhibiting OGT both *in vitro* and in cellular contexts (Hammel et al., 2024).

To enhance inhibition efficacy, bisubstrate inhibitors have been optimized. For example, Goblin1 and Goblin2 synergistically occupied donor and acceptor sites by linking UDPs to peptides via short chains, but were dependent on membrane-penetrating peptide modifications to enhance cell permeability (Borodkin et al., 2014). Further refinement yielded S-linked UDP-peptide analogs, where thioether linkages replaced oxygen-based bonds, improving stability and antiplane conformations for enhanced inhibitory efficiency (Rafie et al., 2018). Recently, a series of bisubstrate ether-linked uridine-peptide conjugates derived from uridine and GlcNAc were developed as OGT inhibitors (Makwana et al., 2021).

At the same time, breakthroughs have been made in the field of small molecule inhibitors through high-throughput screening and natural product mining (Alteen et al., 2020). Among the O-GlcNAc substrate mimic inhibitor (OSMI) series, OSMI-1 (Ortiz-Meoz et al., 2015), its derivatives OSMI-2 (Itkonen et al., 2019), and OSMI-4 (Loi et al., 2020) exhibited nanomolar affinity and superior cell permeability, serving as optimal tool compounds. In rat cortical neurons, Alloxan, BXZ2, and OSMI-1 reduced O-GlcNAcylation levels and enhanced mTOR-dependent autophagy, implicating OGT inhibition in neuronal regulation (Rahman et al., 2020). A spinal cord–like bioprinted scaffold incorporating OSMI-4 promoted neuronal differentiation of NSCs, enabling effective spinal cord injury repair (Liu et al., 2022). The natural product derivative L01 specifically bound the UDP pocket via hydrogen-bond networks, offering a novel pathway for natural product-based inhibitor development (Liu et al., 2017b).

Tethering *in situ* click chemistry identified APNT and APBT as cell-permeable OGT inhibitors that reduced cellular O-GlcNAcylation without compromising viability (Wang et al., 2017). The peptidomimetic LQMED330 demonstrated *in vitro* human OGT inhibition but exhibited potential toxicity (Albuquerque et al., 2020). Recently, homogeneous time-resolved fluorescence-based high-throughput screening identified Bardoxolone, a novel OGT inhibitor binding the TPR and N-terminal domains of OGT (Wu et al., 2023b). Machine learning classification models and molecular dynamics simulations suggested the nonsteroidal anti-inflammatory drug celecoxib as a potential OGT inhibitor (Azevedo et al., 2024).

### O-GlcNAcase inhibitors

The development of OGA inhibitors has evolved through multiple generations, progressing from early broad-spectrum tool compounds to highly selective therapeutic candidates. Inhibitors with carbohydrate backbones have always dominated, with PUGNAc, the first synthetic inhibitor, showing nanomolar-level inhibitory activity but insufficient selectivity for lysosomal β-hexosaminidase (Haltiwanger et al., 1998). Notably, PUGNAc ameliorated Alzheimer’s disease progression by promoting non-amyloidogenic processing (Chun et al., 2015). Streptozotocin, a non-competitive chemical blocker of OGA, suppressed growth hormone and insulin secretion with poor inhibitory activity and significant off-target effects (Liu et al., 2002). Similar to the dose-response of streptozotocin, Alloxan not only inhibited OGT, but also was an effective inhibitor of pancreatic OGA, but had a poor effect on brain OGA (Lee et al., 2006).

These limitations spurred breakthroughs in the NAG-thiazoline series. NAG-thiazoline achieved balanced inhibition by mimicking the distorted chair conformation of the oxazoline intermediate via its thiazoline ring, while NButGT enhances HexB selectivity 1500-fold by introducing a propyl side chain to occupy a hydrophobic pocket unique to human OGA (Macauley et al., 2005). NButGT’s cell permeability enabled it to reduce Aβ plaque burden, neuroinflammation, and memory deficits in Alzheimer’s disease mouse models (Kim et al., 2013). Thiamet-G, derived from NButGT by replacing the terminal methyl group with an amino moiety, further optimized the active site geometry, achieving a Ki of 21 nM and unprecedented HexB selectivity (37,000-fold) (Yuzwa et al., 2008). It emerged as the first preclinical candidate to significantly reduce tau aggregation in Alzheimer’s disease. Thiamet-G was widely used to probe cerebral O-GlcNAc functions, persistently elevating O-GlcNAcylation in Alzheimer’s disease transgenic mice (Alghusen et al., 2024) and reducing phosphorylation at tau Thr231/Ser396 residues in PC12 cells, rat cortex, and hippocampus (Yuzwa et al., 2008). In Parkinson’s disease models, Thiamet-G impaired autophagic flux (Wani et al., 2017), regulated motor learning response (Shao et al., 2022), and inhibited α-syn uptake and propagation (Tavassoly et al., 2021). Its derivative MK-8719, with superior CNS penetration, has entered Phase I clinical trials for tauopathies (Selnick et al., 2019). In Alzheimer’s disease brains, MK-8719 elevated O-GlcNAc levels, reduced pathological tau and Aβ, mitigated brain atrophy, and alleviated neurodegeneration (Rudrawar and Ryan, 2019; Wang et al., 2020).

Another landmark inhibitor, GlcNAcstatins, was based on the imidazole ring structure of the natural product NAGstatin and achieved nanomolar inhibition and over 900,000-fold hexosaminidase selectivity through side chain modification. Its design strategy of covalently targeting the human OGA active site cysteine provided new ideas for high selectivity. An *in vitro* study showed that GlcNAcstatins increased the level of O-GlcNAc in human HEK293 and SH-SY5Y neuroblastoma cell lines (Dorfmueller et al., 2006). α-GlcNAc thiolsulfonates selectively bound short OGA via disulfide bonds, achieving 4–9 fold selectivity over long OGA and HexB (Kim et al., 2007). Additional OGA inhibitors, including 1,2,3-triazole derivatives (Igual et al., 2019), thiosugar-naphthalimides (Shen et al., 2018), and bicyclic selenazolidines (Velueta-Viveros et al., 2022), expanded the therapeutic arsenal.

Recent advances focused on tau pathology and neurodegeneration. Diaminocyclopentane-derived inhibitors elevated neuronal O-GlcNAc without toxicity (Weber et al., 2022). SEG28019 reduced tau phosphorylation at Ser396/Ser404 in dorsal root ganglion neurons (Mellone et al., 2013). Thiamme2-G rescued cognitive deficits by attenuating tau hyperphosphorylation in mice (Pan et al., 2021). ASN90, an orally bioavailable inhibitor, suppressed brain OGA activity, lowered phosphorylated tau at Ser396, and improved phenotypes in APP/PS1 mice (Permanne et al., 2022). Highly substituted diaminocyclopentane-l-lysine adducts became potential drugs for the development of anti-tau phosphorylation reagents due to their ease of preparation and significant biological activity (Weber et al., 2023, 2024). Non-carbohydrate 4-(arylethynyl)piperidine derivatives enhanced O-GlcNAcylation, reduced tau phosphorylation (Ser199, Thr205, and Ser396), and rescued cognitive deficits in APP/PS1 mice (Cheng et al., 2024b). Clinically, Ceperognastat (LY3372689), an oral, CNS-penetrant OGA inhibitor, demonstrated over 95% brain OGA occupancy in Phase I trials, supporting advancement to Phase II (Kielbasa et al., 2024).

Emerging research on O-GlcNAcylation has unveiled novel therapeutic avenues for neurodegenerative diseases. Significant progress has been made in developing regulatory strategies, including metabolic pathway activation via GlcN supplementation and precision modulation of enzymatic activity through OGT/OGA inhibitors. However, translating these strategies into effective clinical treatments faces substantial hurdles. A major barrier lies in delivering these agents effectively to the brain across the blood-brain barrier, which severely restricts the passage of many potential drugs. Furthermore, the selectivity of these drugs for different tissues and organs also poses new challenges for intervening in neurodegenerative diseases. Although these approaches have demonstrated efficacy in animal models of neurodegenerative diseases, only a limited number of candidates, such as MK-8719 and Ceperognastat, have progressed to early-stage clinical trials. To date, regulatory agencies, including the U.S. Food and Drug Administration and the China Food and Drug Administration, have not approved any O-GlcNAc-based therapies for neurodegenerative disorders. Future research must prioritize optimizing drug-targeting specificity and safety profiles. Concurrently, accelerating clinical translation to validate the therapeutic efficacy of these strategies in human populations will be pivotal to overcoming current bottlenecks and realizing their potential in combating neurodegeneration.

## Limitations

Several limitations remain in our review. First, the majority of experimental evidence included in this synthesis is derived from cellular and animal models, lacking sufficient clinical studies to validate how these phenomena and mechanisms operate specifically in humans. Second, research in this field, particularly in developing targeted therapies against O-GlcNAcylation, remains in relatively early stages. There is very limited data on the efficacy and safety of O-GlcNAcylation modulators in the treatment of neurodegenerative diseases. Third, while our review identified bidirectional effects and some contradictory findings regarding O-GlcNAcylation, the inherent complexity of these mechanisms means our literature-based analysis alone cannot definitively explain the specific causes underlying these dual regulatory roles. Finally, our search strategy was restricted to English-language journal papers, potentially excluding relevant studies published in other languages or publication forms.

## Conclusion and Perspective

**Figure 10:**
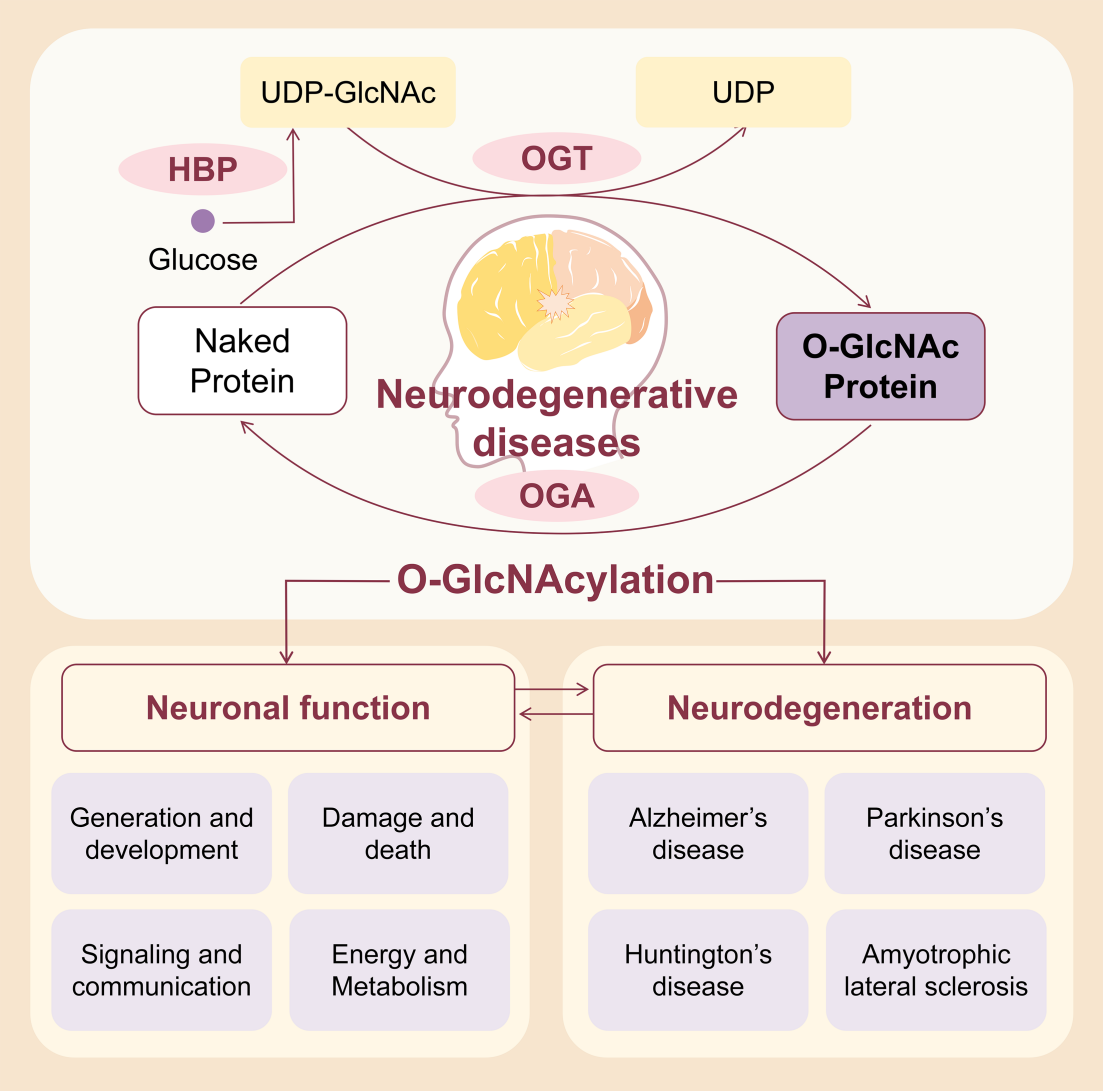
O-GlcNAcylation in neurodegenerative diseases. O-GlcNAcylation regulated by OGT and OGA plays an important role in neurodegenerative diseases. On the one hand, O-GlcNAcylation regulates neuronal pathological and physiological functions. On the other hand, O-GlcNAcylation regulates various diseases such as AD, PD, HD, and ALS. Created with Word Processing System Presentation (version 12.1.0.19302). AD: Alzheimer’s disease; ALS: amyotrophic lateral sclerosis; HBP: hexosamine biosynthetic pathway; HD: Huntington’s disease; O-GlcNAc: N-acetylglucosamine; OGA: O-GlcNAcase; OGT: O-GlcNAc transferase; PD: Parkinson’s disease; UDP: uridine diphosphate.

O-GlcNAcylation, a dynamic and reversible post-translational modification, has emerged as a focal point in neurodegenerative disease research. By modulating protein function, signaling pathways, and metabolic states, it profoundly influences neuronal development, survival, synaptic plasticity, and pathological progression. This review synthesizes current evidence on the multifaceted roles of O-GlcNAcylation across Alzheimer’s disease, Parkinson’s disease, amyotrophic lateral sclerosis, Huntington’s disease, and related neurodegenerative disorders, unraveling its complex regulatory landscape from molecular mechanisms to clinical transformation (**Figure 10**). However, there are still many contradictions and challenges in the research of this field. In the future, breakthroughs need to be made in technology, mechanisms, and treatment strategies in order to promote a substantial leap from basic discoveries to clinical applications.

At the core of neuronal physiology, O-GlcNAcylation maintains functional-metabolic equilibrium by balancing OGT and OGA activities. The proliferation and differentiation of NSCs, axon guidance, and synaptic transmission all depended on the O-GlcNAcylation of specific proteins. For example, O-GlcNAcylation of Notch1 stabilized the hippocampal NSCs pool to sustain adult neurogenesis (Chen et al., 2021). OGT was enriched in the PSD of excitatory synapses and promoted O-GlcNAcylation on a variety of proteins after neuronal stimulation, contributing to dendritic spine maturation (Lagerlöf et al., 2017). In terms of energy metabolism, O-GlcNAcylation regulated cellular nutrition, coordinated mitochondrial function, and brain glucose metabolism. The O-GlcNAcylation of hexokinase 1, for instance, enhanced its mitochondrial association to boost glycolytic flux (Wang et al., 2024). From embryonic neurogenesis and axonal elongation to synaptic formation and programmed cell death, O-GlcNAcylation permeated every phase of neuronal existence, suggesting its critical role as a guardian of homeostasis.

In neurodegenerative diseases, dysregulation of O-GlcNAcylation exhibits strong correlations with pathological hallmarks, yet its regulatory effects and mechanisms diverge across disease types. In Alzheimer’s disease, tau hyperphosphorylation and Aβ deposition were tightly linked to reduced O-GlcNAcylation. While O-GlcNAcylation of tau inhibited its phosphorylation and fibrillization, the primary sites of this reciprocal modification were poorly defined, and their stoichiometric relationships were unquantified. Similarly, O-GlcNAcylation of APP promoted non-amyloidogenic processing to limit Aβ production, yet the specific peptide segments regulated by this modification were uncharacterized. Parkinson’s disease studies revealed a more complex bidirectional management. O-GlcNAcylation of α-syn, particularly at Thr72 and Ser87 within the NAC domain, effectively blocked its pathological aggregation. Paradoxically, excessive O-GlcNAc levels in dopaminergic neurons inhibited TH phosphorylation, reducing dopamine synthesis and exacerbating Parkinson’s disease progression. Similar contradictions emerged in Huntington’s disease. Restoring O-GlcNAcylation of nucleoporin NUP62 alleviated mHTT aggregation-induced nucleocytoplasmic transport defects, whereas elevated O-GlcNAc impaired autophagy, promoting toxic protein accumulation. In amyotrophic lateral sclerosis, O-GlcNAcylation exerted dual protective roles by suppressing TDP-43 aggregation and maintaining neurofilament homeostasis, collectively mitigating neurodegeneration.

Although these findings provide a theoretical basis for the role of O-GlcNAcylation in regulating neurodegenerative diseases, current studies still face multiple contradictions and limitations. First, the bidirectional regulatory nature of O-GlcNAcylation complicates its pathological interpretation and poses challenges for clinical applications. For instance, O-GlcNAcylation exerted protective effects on mitochondrial bioenergetics, network integrity, and viability (Pinho et al., 2019), yet O-GlcNAcylation of Drp1 promoted mitochondrial fission and dysfunction (Park et al., 2021c). Whole O-GlcNAc deficiency impaired cognition and memory in Alzheimer’s disease models (Chen et al., 2021), while loss of O-GlcNAcylation of cyclic AMP-response element binding protein enhanced long-term memory consolidation (Rexach et al., 2012). This contradiction likely stems from the divergent functional outcomes between whole O-GlcNAcylation and protein-specific modifications, while the precise mechanisms remain to be fully elucidated. Second, existing technologies inadequately resolve the spatiotemporal heterogeneity of O-GlcNAcylation. Although mass spectrometry identifies O-GlcNAc sites, its sensitivity for low-abundance proteins or transient modifications remains insufficient, and tools for real-time monitoring of O-GlcNAc dynamics in living cells are lacking. Additionally, the reductionist limitations of disease models are apparent. The transgenic animal models often overexpress single mutant proteins (e.g., tauP301L), failing to simulate the chronic progression characteristics of human diseases and microenvironment interactions.

At the translational medicine level, O-GlcNAc-targeted therapeutic strategies face significant clinical challenges. A major hurdle is the insufficient selectivity of current OGT/OGA inhibitors. Although OSMI inhibitors have established OGT inhibitory efficacy, their uncertain isoform selectivity may lead to off-target effects. Thiamet-G, a highly selective OGA inhibitor, shows promise in reducing tau phosphorylation in Alzheimer’s disease models, yet its efficacy in other neurodegenerative contexts remains underexplored, with development largely confined to preclinical stages. Although OGA inhibitors like MK-8719 have entered clinical trials, the presence of chronic toxicity with their long-term use remains to be verified. Furthermore, nutritional interventions (e.g., GlcN supplementation) similarly lack rigorous evaluation of long-term metabolic consequences. Excessive and sustained elevation of O-GlcNAc may exacerbate insulin resistance, resulting in specific risks such as diabetes. Collectively, these limitations converge on a central question: How can we achieve precise modulation of O-GlcNAcylation in complex biological systems to balance their neuroprotective benefits against potential toxicity?

Looking ahead, technological innovation and interdisciplinary integration will be pivotal to overcoming current bottlenecks. In foundational research, the development of highly sensitive and high-resolution modification detection technologies is imperative. Integrating single-cell proteomics with spatial transcriptomics could map O-GlcNAcylation patterns across distinct brain regions and neuronal subpopulations. Artificial intelligence offers transformative potential. The machine learning models can predict modification sites and their functional impacts, while multi-omics data integration enables the construction of O-GlcNAc regulatory networks, systematically uncovering its crosstalk with phosphorylation, ubiquitination, and other post-translational modifications.

In summary, research on O-GlcNAcylation stands at a critical juncture, transitioning from fundamental exploration to clinical intervention. Its multidimensional role in neurodegenerative diseases provides both rich therapeutic targets and challenges posed by the complexity of the mechanism. Future research needs to be driven by technological innovation and oriented toward precision medicine in order to sustain breakthroughs in areas such as analyzing modification dynamics, revealing disease heterogeneity, and developing smart therapies.

## Data Availability

*Not applicable.*
